# Intravital imaging technology guides FAK-mediated priming in pancreatic cancer precision medicine according to Merlin status

**DOI:** 10.1126/sciadv.abh0363

**Published:** 2021-09-29

**Authors:** Kendelle J. Murphy, Daniel A. Reed, Claire Vennin, James R. W. Conway, Max Nobis, Julia X. Yin, Cecilia R. Chambers, Brooke A. Pereira, Victoria Lee, Elysse C. Filipe, Michael Trpceski, Shona Ritchie, Morghan C. Lucas, Sean C. Warren, Joanna N. Skhinas, Astrid Magenau, Xanthe L. Metcalf, Janett Stoehr, Gretel Major, Ashleigh Parkin, Romain Bidanel, Ruth J. Lyons, Anaiis Zaratzian, Michael Tayao, Andrew Da Silva, Lea Abdulkhalek, Australian Pancreatic Genome Initiative (APGI), Australian Pancreatic Cancer Matrix Atlas (APMA), Anthony J. Gill, Amber L. Johns, Andrew V. Biankin, Jaswinder Samra, Sean M. Grimmond, Angela Chou, Jacky G. Goetz, Michael S. Samuel, J. Guy Lyons, Andrew Burgess, C. Elizabeth Caldon, Lisa G. Horvath, Roger J. Daly, Nikolaj Gadegaard, Yingxiao Wang, Owen J. Sansom, Jennifer P. Morton, Thomas R. Cox, Marina Pajic, David Herrmann, Paul Timpson

**Affiliations:** 1 Garvan Institute of Medical Research and The Kinghorn Cancer Centre, Cancer Division, Sydney, NSW 2010, Australia.; 2 St. Vincent’s Clinical School, Faculty of Medicine, UNSW Sydney, Sydney, NSW 2010, Australia.; 3 Oncode Institute, Division of Molecular Pathology, The Netherlands Cancer Institute, Plesmanlaan 121, 1066 CX Amsterdam, Netherlands.; 4 Turku Bioscience Centre, University of Turku and Åbo Akademi University, FI-20520 Turku, Finland.; 5 Sydney Medical School, University of Sydney, Sydney, NSW 2006, Australia.; 6 NSW Health Pathology, Department of Anatomical Pathology, Royal North Shore Hospital, NSW 2065, Australia.; 7 Cancer Diagnosis and Pathology Research Group, Kolling Institute of Medical Research, NSW 2064, Australia.; 8 Institute of Cancer Sciences, Wolfson Wohl Cancer Research Centre, Institute of Cancer Sciences, University of Glasgow, Glasgow, UK.; 9 West of Scotland Pancreatic Unit, Glasgow Royal Infirmary, Glasgow, UK.; 10 Department of Surgery, Royal North Shore Hospital, Sydney, NSW 2065, Australia.; 11 University of Melbourne Centre for Cancer Research, Victorian Comprehensive Cancer Centre, 305 Grattan Street, Melbourne, VIC 3000, Australia.; 12 Department of Anatomical Pathology, SydPath, Darlinghurst, NSW 2010, Australia.; 13 INSERM UMR, Tumour Biomechanics, Strasbourg, France.; 14 Université de Strasbourg, Strasbourg, France.; 15 Fédération de Médecine Translationnelle de Strasbourg (FMTS), Strasbourg, France.; 16 Centre for Cancer Biology, SA Pathology and University of South Australia, SA, Australia.; 17 Adelaide Medical School, Faculty of Health and Medical Sciences, University of Adelaide, Adelaide, SA, Australia.; 18 Dermatology, Sydney Medical School, University of Sydney, Camperdown, NSW, Australia.; 19 Cancer Services, Royal Prince Alfred Hospital, Camperdown, NSW, Australia.; 20 Centenary Institute, The University of Sydney, Camperdown, NSW, Australia.; 21 ANZAC Research Institute, Sydney, NSW 2139, Australia.; 22 Faculty of Medicine and Health, Concord Clinical School, University of Sydney, Sydney, NSW 2000, Australia.; 23 Medical Oncology, Chris O’Brien Lifehouse, Camperdown, NSW 2006, Australia.; 24 Cancer Program and Department of Biochemistry and Molecular Biology, Monash Biomedicine Discovery Institute, Monash University, Melbourne, VIC 3800, Australia.; 25 James Watt School of Engineering, University of Glasgow, Glasgow, UK.; 26 Department of Bioengineering and Institute of Engineering in Medicine, University of California, San Diego, La Jolla, CA, USA.; 27 Cancer Research UK Beatson Institute, Glasgow, UK.

## Abstract

Pancreatic ductal adenocarcinoma (PDAC) is a highly metastatic, chemoresistant malignancy and is characterized by a dense, desmoplastic stroma that modulates PDAC progression. Here, we visualized transient manipulation of focal adhesion kinase (FAK), which integrates bidirectional cell-environment signaling, using intravital fluorescence lifetime imaging microscopy of the FAK-based Förster resonance energy transfer biosensor in mouse and patient-derived PDAC models. Parallel real-time quantification of the FUCCI cell cycle reporter guided us to improve PDAC response to standard-of-care chemotherapy at primary and secondary sites. Critically, micropatterned pillar plates and stiffness-tunable matrices were used to pinpoint the contribution of environmental cues to chemosensitization, while fluid flow–induced shear stress assessment, patient-derived matrices, and personalized in vivo models allowed us to deconstruct how FAK inhibition can reduce PDAC spread. Last, stratification of PDAC patient samples via Merlin status revealed a patient subset with poor prognosis that are likely to respond to FAK priming before chemotherapy.

## Teaser

Intravital imaging guides a personalized medicine approach to target mechanoreciprocity in pancreatic cancer.

## INTRODUCTION

Despite improvements in drug discovery and therapeutic efficacy, pancreatic ductal adenocarcinoma (PDAC) remains one of the most lethal cancers worldwide (*1*). While the addition of nab-paclitaxel (Abraxane) to traditional gemcitabine monotherapy improved median patient survival by ~6 weeks, the overall 5-year survival rate has remained virtually stagnant at <10% for the past four decades (*1*, *2*). A substantial proportion of patients with PDAC present with metastatic disease at the time of diagnosis, and once metastasized, the 5-year survival further decreases to a dismal 3% (*1*, *2*). PDAC formation and progression are accompanied by the deposition of a dense desmoplastic extracellular matrix (ECM) or stroma that increases the biomechanical force load on cancer cells and is thought to both promote and restrict disease advancement (*3*–*5*). While complete ablation of stromal components has been shown to reduce survival, recent studies focusing on the transient targeting of the stroma have proven promising because of the subtle alterations that this approach can induce on tissue architecture, stiffness, or mechanoplasticity in PDAC (*3*, *4*, *6*–*11*). Hence, there is a need to deconstruct the clinical benefits of transient versus chronic stromal manipulation while optimizing epithelial targeting before chemotherapy to obtain the maximal outcome in this disease.

Focal adhesion kinase [FAK or PTK2 (protein tyrosine kinase 2)] is a ubiquitously expressed nonreceptor tyrosine kinase that mediates communication between cells and their environment (*12*). It regulates bidirectional integrin-mediated “inside-out and outside-in signaling” (*13*, *14*) and is often hyperactivated and overexpressed in multiple aggressive cancers (*12*, *13*, *15*). The reciprocal interactions between cancer cells and their surrounding stromal architecture drive signaling pathways that have been shown to promote matrix remodeling; induce tissue stiffness; and accelerate cell proliferation, cell survival, and disease progression (*14*, *16*–*18*). Thus, understanding and optimizing the individual responses of cancer and stromal cells to FAK inhibition (hereafter referred to as epithelial and stromal targeting, respectively), rather than epithelial cell targeting alone, may provide insights into potential therapeutic regimens to combat this aggressive disease at the intersection of tumor-stromal cross-talk.

An important step in the clinical management of disease is the ability to match a selective drug target with a specific molecular signature that may predict drug sensitivity (*19*). The canonical tumor suppressor, Merlin (*NF2*), has been shown to regulate the function and activity of cell surface receptor tyrosine kinases, modulating “outside-in” signaling to control contact-induced inhibition of proliferation and tumor progression in various cancer types (*20*–*22*). In preclinical models (*20*) and clinical studies (*21*) in a diverse panel of cancers, enhanced efficiency of FAK inhibitors (FAKi) was observed in tumors harboring reduced Merlin levels; however, a correlation between Merlin expression and FAKi sensitivity in PDAC remains to be assessed.

Intravital imaging provides insight into how epithelial and stromal cells behave in the native tumor microenvironment, allowing optimization of therapeutic response in real time (*23*–*26*). Here, we use well-validated primary pancreatic cancer (PC) cells derived from the aggressive metastatic KPC (Pdx1-Cre, LSL-Kras^G12D/+^, LSL-Trp53^R172H/+^) mouse model of PDAC, which retain their invasive and metastatic capacity ex vivo (*18*, *26*–*29*), and patient-derived cell lines (PDCLs) from the Australian Pancreatic Genome Initiative (APGI) to dissect and optimize the benefits of stromal and epithelial targeting in this disease (*18*, *30*–*32*). We used the FAK-based Förster resonance energy transfer (FRET) biosensor (*33*) or the Fluorescent ubiquitination-based cell cycle indicator (FUCCI) cell cycle reporter (*34*) for in vivo imaging to monitor dynamic changes in FAK activity and gemcitabine/Abraxane-induced effects on KPC cell cycle distribution, respectively. This imaging approach allowed us to optimize treatment response at both primary and secondary sites in live tissue. Following pulsed FAKi priming, we reveal subtle changes in the ultrastructure of the ECM architecture along with changes in both FAK activity and cell cycle distribution following gemcitabine/Abraxane chemotherapy in live primary tumors and at secondary sites within the liver. Using micropatterned pillar plates and stiffness-tunable matrices, we elucidate how the mechanics of the microenvironment contribute to this chemosensitization. In parallel, cell streaming, personalized organotypic assays, and fluid flow–induced shear stress assessment allowed us to deconstruct and streamline stromal and epithelial FAK inhibition before chemotherapy, highlighting the sensitivity of tumor cells to pulsed FAK inhibition during transit, which may ultimately prevent seeding and colonization in the metastatic niche. Using long-term orthotopic assessment of metastatic burden and survival via tailored targeting of PDCLs with high versus low FAK signatures, we reveal a personalized response to transient stromal priming via FAK inhibition relative to inherent “inside-out” FAK signaling axis status. Furthermore, following assessment of a graded response to FAK inhibition, we evaluate Merlin expression levels in the context of high versus low FAK status in patient settings via the International Cancer Genome Consortium (ICGC) cohort (*30*–*32*). The capacity to further stratify individual patient response to FAK inhibition using low Merlin expression as a potential positive biomarker may provide a promising guide in the clinical management of PDAC treatment. Together, we present preclinical and clinically relevant data that streamline stromal and epithelial FAK inhibition to guide ongoing FAK-based clinical applications in both the primary and metastatic settings of PDAC. Furthermore, we reveal intertumoral heterogeneity within pancreatic patient tumors that could tailor FAKi priming before standard-of-care chemotherapy in this disease to significantly ablate tumor progression based on Merlin status.

## RESULTS

### FAK expression and activity increase with disease progression in the KPC mouse model of PDAC and correlate with poor patient prognosis

**Fig. 1.**
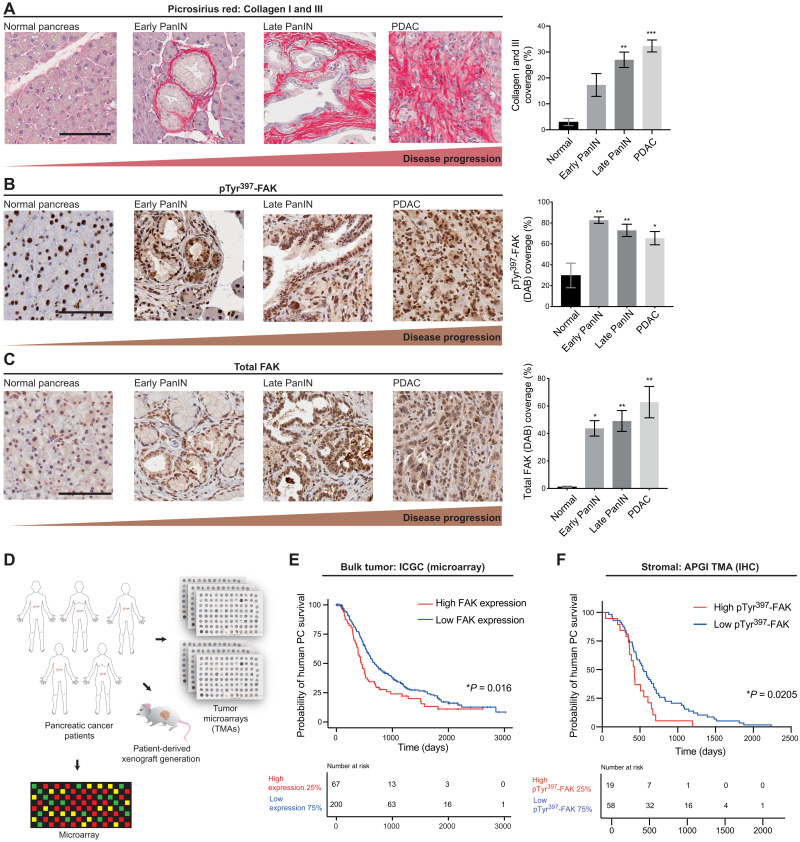
ECM abundance and FAK expression and activity increase throughout PDAC progression, providing a rationale to target tumor-ECM feedback in PC. (**A** to **C**) Representative images of normal pancreas, early Pancreatic intraepithelial neoplasia (PanIN), late PanIN, and PDAC tissue (scale bars, 100 μm) and quantification of collagen I/III coverage (Picrosirius red) (A), phosphorylated (pTyr^397^) FAK (DAB) (B), and total FAK expression (DAB) (C). *n* ≥ 3 mice per stage and *n* ≥ 3 fields of view (FOVs) per stage per mouse. Results: means ± SEM. *P* values were determined using an ordinary one-way analysis of variance (ANOVA) with Tukey correction for multiple comparisons, and significance is compared to normal pancreas. (**D**) Schematic representation of patient-derived xenograft (PDX) and parallel TMA establishment. (**E**) Kaplan-Meier analysis of human PC disease survival based on FAK expression (microarray) with high FAK (red) versus low FAK (blue) in the ICGC cohort. (**F**) Kaplan-Meier analysis of deceased human PC patient survival with low Picrosirius red staining and high pTyr^397^-FAK (red) versus low pTyr^397^-FAK (blue) in the APGI cohort. Patients who died of surgical complication or whose deaths were non–cancer-related were excluded from the analysis. Significance was determined using a Kaplan-Meier analysis of survival compared using a log-rank test. *P < 0.05, **P < 0.01, and ***P < 0.001.

To evaluate the levels of FAK expression and activity during PDAC progression, we initially analyzed autochthonous KPC mouse tumors at different stages of disease progression using Picrosirius red collagen I and III costaining, coupled with assessment of total and phosphorylated (pTyr^397^) FAK by immunohistochemistry (IHC). We found that the expression and activity of FAK increased through PDAC disease progression, concomitant with enhanced fibrosis (Fig. 1, A to C, and fig. S1, A to C). This was confirmed by immunofluorescence costaining of α–smooth muscle actin (αSMA) (stroma) or E-cadherin (epithelial) and pTyr^397^-FAK (fig. S1, D and E, respectively). In line with this, bulk assessment of FAK (*PTK2*) expression levels in patients with PDAC from the ICGC (267 samples) demonstrated that high FAK expression (top 25%) correlated with poor survival (Fig. 1, D and E). Moreover, IHC analysis of human biopsy tumor microarrays (TMAs) of a cohort of 231 patient samples from the APGI further revealed that high stromal pTyr^397^-FAK was also predictive of poor clinical survival in patients with early fibrosis, as assessed via costaining with Picrosirius red (Fig. 1F and fig. S1, B and C). When quantifying fibrosis via Picrosirius red staining in surgical samples of patients with PDAC from the stroma-focused Australian Pancreatic Cancer Matrix Atlas (APMA) cohort, we observed a significant increase in fibrosis in patients with PDAC who received presurgical neoadjuvant gemcitabine/Abraxane chemotherapy compared to treatment-naïve patients (fig. S1J). This indicates that an early fibrotic stromal reaction is one of the first responses to chemotherapy in this disease. To provide a potential avenue for optimized FAK targeting regimens and improved outcomes in this multifaceted and highly heterogeneous disease, we sought to first dissect the role that pharmacological FAK inhibition could have on stroma and epithelium in the complex microenvironment of pancreatic tumors. As FAK expression and activity levels can vary both at the intertumoral patient level and intratumoral levels within the tumor’s stromal and epithelial compartments (*30*–*32*), tailoring treatment regimens depending on individual tumor status could allow for a personalized approach to FAK targeting in PC.

### Deconstructing the benefits of epithelial and stromal FAK inhibition in PDAC

**Fig. 2.**
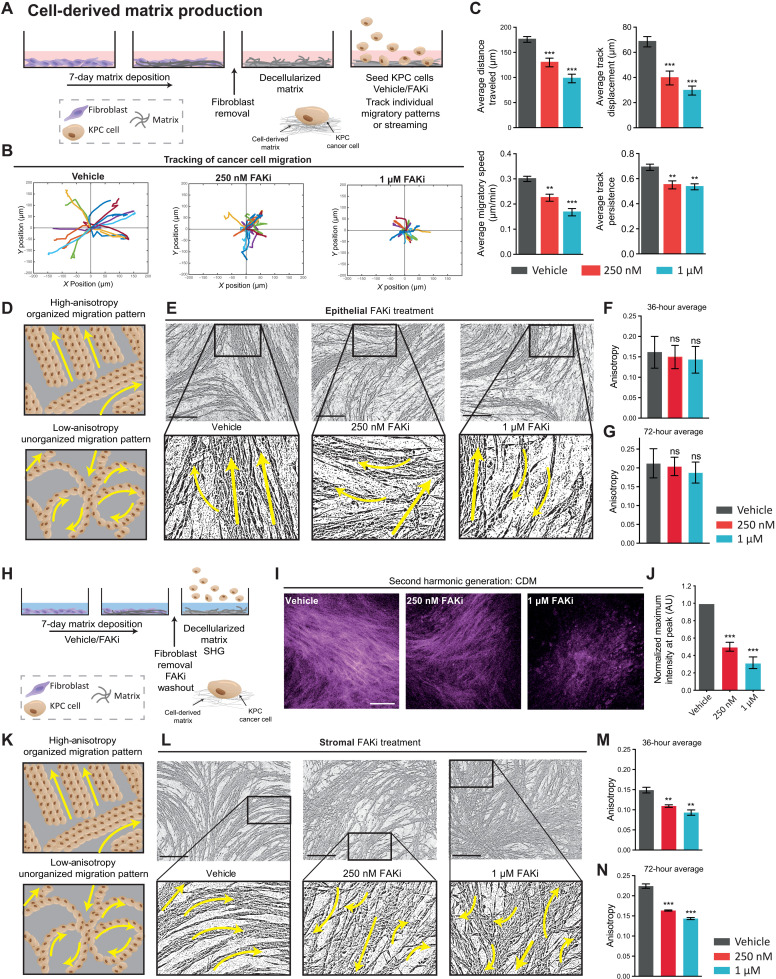
Effect of epithelial versus stromal FAK inhibition on single and collective KPC cancer cell migration. (**A**) Schematic of CDM generation, decellularization, and KPC cell seeding. (**B**) Representative tracks of KPC cell migration on CDMs upon treatment with vehicle or FAKi over 8 hours. (**C**) Quantification of KPC cell average distance traveled, displacement from starting position, speed, and track persistence over 8 hours. (**D**) Schematic of organized (high anisotropy, top) and unorganized cell migration (low anisotropy, bottom). (**E** to **G**) Representative binary images of KPC cell streaming 72 hours after seeding treated with vehicle or FAKi during migration (E) (arrows represent stream directionality; scale bars, 200 μm) with quantification of average anisotropy at 36 (F) and 72 hours (G). (**H**) Schematic of CDM production treated with vehicle or FAKi. (**I** and **J**) Representative maximum intensity second harmonic generation (SHG) images in CDMs treated with vehicle or FAKi (I) (scale bar, 100 μm) with quantification of peak SHG signal intensity (J). (**K**) Schematic of organized (high anisotropy, top) and unorganized cell migration (low anisotropy, bottom). (**L** to **N**) Representative binary images of KPC cell streaming 72 hours after seeding on vehicle or FAKi-primed CDMs (L) (arrows represent stream directionality; scale bars, 200 μm) with quantification of average anisotropy at 36 (M) and 72 hours (N). *n* = 3 biological repeats, 3 CDMs per repeat, 3 FOVs per CDM (15 cells per FOV) (C). Results: means ± SEM. *P* values were determined using an ordinary one-way ANOVA with Tukey correction for multiple comparisons. Unless otherwise stated, all significance is compared to vehicle. ns, *P* > 0.05; **P < 0.01, and ***P < 0.001. AU, arbitrary units.

As PDAC is a highly aggressive and metastatic cancer, we initially assessed the direct effects of pharmacological FAK inhibition on cell movement in 2.5-dimensional (2.5D) cell-derived matrices (CDMs) using primary cells lines generated from the KPC model of PC (*27*, *35*, *36*). CDMs were established to provide a high-fidelity ECM substrate and platform to examine the effects of FAK inhibition on coordinated single and collective cell motility in real time (Fig. 2A). Following KPC cell adhesion to decellularized matrix, KPC cells were treated with the FAKi PF-562271 (FAKi; Fig. 2A and fig. S1, F and G) (*12*, *37*), and individual cell tracking demonstrated a significant reduction in average distance traveled, track displacement, migratory speed, and track persistence of cells compared to control settings (Fig. 2, B and C). In addition, assessing cell orientation relative to a wound revealed that this may, in part, be due to a loss of cell polarity upon FAKi treatment indicated by a loss of the cells’ ability to orientate the Golgi body to the leading edge of the cell, as previously described (fig. S1, H and I) (*38*, *39*).

Next, we assessed collective cell migration or “streaming” in this setting, which more faithfully recapitulates tumor cell motility and invasion found in vivo. Here, we used image anisotropy to quantify the coordinated nature of cell migration, which can often facilitate a more efficient mode of invasion, whereby cells collectively move in the same direction and follow a stream of least resistance (see schematic in Fig. 2D) (*18*, *40*, *41*). Image anisotropy analysis was performed over a 72-hour period, where “high anisotropy” indicated collective coordinated migration between cancer cells and “low anisotropy” indicated uncoordinated migration, as recently described (Fig. 2D) (*18*, *42*). This revealed that while FAK inhibition altered individual KPC cell migration on CDMs (Fig. 2, A to C), collective cell movement was not significantly changed when targeting the epithelial tumor cells alone (Fig. 2, D to G, and movie S1).

As well as neighboring cells influencing cancer cell movement, cancer cells also bias their locomotive direction with guidance from external cues such as the local ECM (*43*–*45*). We therefore assessed whether targeting the ECM via stromal FAK inhibition could affect collective cancer cell migration. Here, fibroblasts were treated with FAKi during CDM production (“priming”) before subsequent FAKi washout (Fig. 2H). Following fibroblast removal and FAKi washout, second harmonic generation (SHG) imaging permitted visualization and quantification of cross-linked collagen, demonstrating that short-term stromal targeting with FAKi significantly reduced the ability of fibroblasts to produce mature and cross-linked collagen compared to control [Fig. 2, I and J (confirmed by Picrosirius red staining) and fig. S2, A and B].

Highly aligned linear matrix fibers serve as migratory routes for cells and are well suited to resisting tensile forces in the alignment direction, whereas a disorganized ECM network is thought to compromise cell migration and tissue mechanics. To assess alterations in the architectural patterns of the fibroblast-derived ECM of CDMs, matrices were fixed and stained with Picrosirius red before polarized light imaging. Analysis of the birefringent signal revealed a decrease in total fibrillar collagen content upon treatment with FAKi (fig. S2, C to E). Further analysis of matrix alignment using FibrilTool (*42*) showed a decrease in matrix anisotropy upon FAKi treatment (fig. S2, F and G). Global matrix organization upon FAKi was further assessed using the ImageJ plugin TWOMBLI (*46*), which highlighted notable differences in addition to fibrillar alignment (fig. S2H). Here, while we observed no change in fibrillar curvature (mean change in angle along a fiber; fig. S2I; please also see inset in fibrillar analysis masks in fig. S2H), larger gaps and increased porosity in control matrices were reflected in the higher lacunarity and lower fractal dimension compared to FAKi-treated matrices (fig. S2, J and K). Analysis of individual fiber organization showed a shift from an anisotropic organized matrix, commonly associated with aggressive cancers (*47*), to an isotropic and poorly organized matrix upon treatment with FAKi. Here, we revealed that FAKi during CDM matrix production led to an increase in the normalized number of fiber branches and endpoints and a corresponding decrease in hyphal growth unit (a measure of the number of endpoints per unit length; fig. S2, L to N). Collectively, this analysis shows that stromal FAKi treatment can disrupt distinct aspects of ECM deposition, remodeling, and organization.

To assess whether this change in matrix deposition and remodeling altered subsequent KPC cell migration, KPC cells were next seeded onto control versus FAKi-pretreated or “primed” matrices. Analysis of individual cell movement on FAKi-primed matrices showed no significant change in cell velocity (average speed; fig. S2O), while the directionality of cell movement (average persistence) was significantly reduced for individual cells on FAKi-treated matrices (fig. S2P). Moreover, quantification of KPC cell streaming revealed that on matrices that had been primed with FAKi during early ECM deposition, cell streaming and collective migration of cancer cells were decreased, with cells moving in an uncoordinated and inefficient manner compared to vehicle priming (Fig. 2, K to N, and movie S2). This disruption of outside-in signaling from the ECM indicated an additional dependence of cancer cells on the surrounding stromal microenvironment to coordinate collective cell migration. Collective cell movement is often observed in vivo at the leading edge of tumors (*40*, *41*) and can play a key role in driving directional invasion before metastatic spread. Hence, we opted to mimic this spatially coordinated cancer cell invasion in response to FAKi in live 3D cocultures using organotypic assays (*18*, *26*, *28*, *48*, *49*).

### Transient matrix priming impairs PDAC invasion and is more effective than chronic treatment

**Fig. 3.**
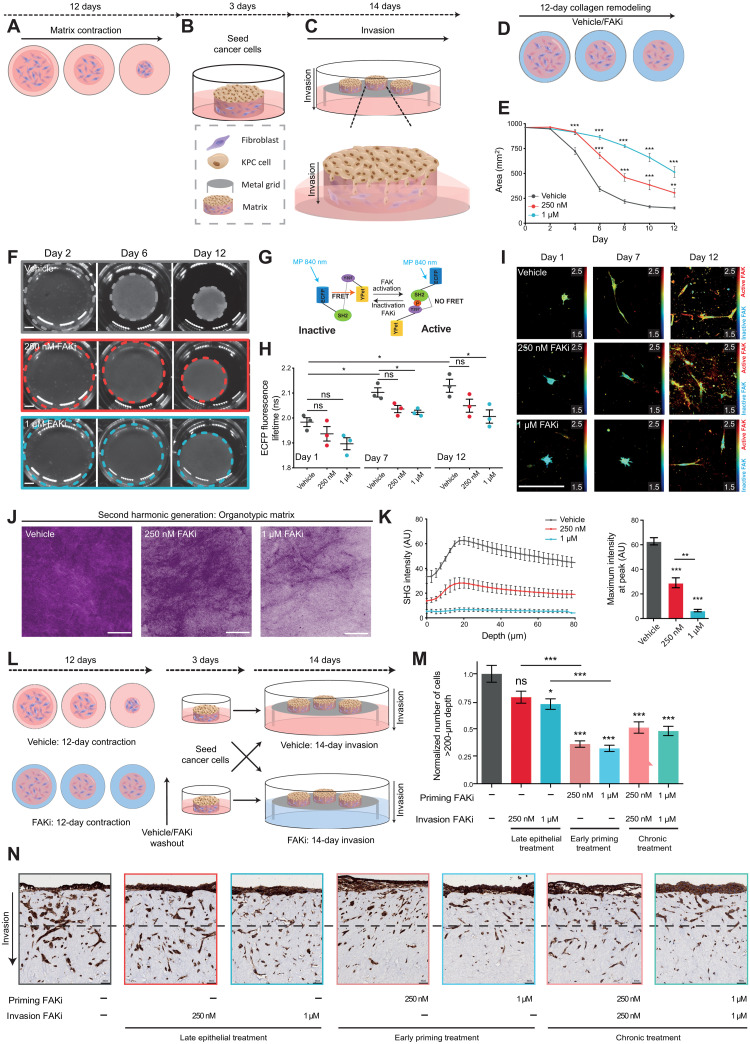
Stromal FAKi priming reduces fibroblast-driven collagen remodeling and KPC cancer cell invasion into organotypic matrices. (**A** to **C**) Schematic of 3D organotypic invasion assay: Fibroblast-driven collagen contraction (A), cancer cell seeding (B), and invasion into the contracted matrix toward the chemotactic gradient, created by air-liquid interface [note the culture medium meniscus (where cells remain in contact with media)] (C). (**D**) Schematic representation of fibroblast-mediated collagen matrix contraction upon vehicle or FAKi priming. (**E** and **F**) Quantification (E) and representative images (F) (scale bars, 5 mm) of matrix contraction over time. (**G**) Schematic of the FAK-FRET biosensor. (**H** and **I**) Quantification (H) and representative intensity-merged maps of ECFP fluorescence lifetime (I) in fibroblasts during matrix remodeling. Scale bar, 100 μm. (**J** and **K**) Representative maximum intensity SHG projections in fibroblast-contracted matrices (J) (scale bars, 100 μm) with quantification of SHG signal intensity (K) at peak (left) and over depth (in μm; right). (**L**) Schematic representation of organotypic invasion assay with late epithelial treatment (during KPC cell invasion), FAKi priming (during matrix contraction), and chronic treatment (during matrix contraction and invasion). (**M** and **N**) Quantification of KPC cell invasion (M) (normalized cell number at >200-μm depth) with representative images of pan-cytokeratin–stained KPC cells (N) (dotted line, 200-μm invasion depth; scale bars, 50 μm). *n* = 3 biological repeats, with three matrices per repeat and three FOVs per matrix (D to N). *n* = 20 cells per condition per replicate (H and I). Results: means ± SEM. *P* values were determined using an ordinary one-way ANOVA with Tukey correction for multiple comparisons. Unless otherwise stated, all significance is compared to vehicle. ns, *P* > 0.05; *P < 0.05, **P < 0.01, and ***P < 0.001.

Organotypic invasion assays (Fig. 3, A to C) enable the assessment of reciprocal interactions between cancer cells and fibroblasts in a 3D collagen-rich environment (*18*, *26*, *28*, *48*, *49*). Here, a 3D-fibrillar collagen matrix is remodeled, cross-linked, and contracted over 12 days by fibroblasts, allowing us to readily assess stromal targeting effects in this disease (see schematic in Fig. 3A). Once formed, PDAC cells are seeded on top of this matrix and then exposed to an air-liquid interface from which they can readily interact with live fibroblasts and the ECM during invasion toward a chemotactic gradient (Fig. 3, B and C). In this way we can understand and deconstruct the stromal versus epithelial responses to FAKi treatment. Fibroblast-driven contraction in the presence of FAKi or vehicle revealed a decrease in matrix contraction (Fig. 3, D to F). Using multiphoton-based fluorescence lifetime imaging microscopy (FLIM)–FRET of a reversible FAK biosensor expressed in the stromal cells (*33*), we could also visualize FAK activation status within these matrices in real time during this process (Fig. 3, G to I). Here, low FAK activity in fibroblasts is displayed in the fluorescence lifetime maps as “cold” blue/green colors indicative of shorter lifetimes, whereas high FAK activity is represented by longer lifetimes and “warm” yellow/red colors (Fig. 3I). Using FLIM-FRET imaging to assess target validation during collagen contraction revealed an increase in FAK activity over the course of matrix remodeling, confirming a critical role for FAK in this process, which was reduced in response to FAKi in this live 3D setting (compare vehicle at days 1, 7, and 12 progressively increasing in lifetime/activity, which was impaired under FAKi conditions; Fig. 3, H and I). The reduced contraction of matrices was independent of changes to fibroblast survival or proliferation during this time, as assessed using cleaved caspase-3 (CC3) and Ki67 IHC staining within matrices (fig. S3, A and B). Furthermore, SHG imaging demonstrated that FAKi altered the ultrastructure of the ECM in this context by reducing collagen cross-linking and remodeling (Fig. 3, J and K, and movie S3), which was confirmed with Picrosirius red–based polarized light imaging (fig. S3, C to G) (*50*) and gray-level co-occurrence matrix (GLCM) analysis (fig. S3, H to K) (*47*), which both confirmed the reduced birefringence and organization of the matrix after FAK inhibition, respectively. Quantification of raw high, medium, and low birefringent Picrosirius red signal (indicative of a range from mature to immature collagen fibers) showed a significant decrease in low birefringent signal (indicative of immature collagen fibers) upon FAKi treatment, suggesting that FAKi could also reduce new matrix production, deposition, and early remodeling of nascent fibers by the fibroblasts in addition to the decrease in established cross-linking that we see with SHG imaging (fig. S3E). Last, biomechanical measurements also revealed that FAKi reduced matrix stiffness as measured by compression and shear rheology analysis (fig. S3, L and M) (*51*).

Given the effects on ECM deposition, remodeling, and stiffness upon FAKi priming, we next assessed whether transient matrix manipulation to alter outside-in signaling was sufficient to impair cancer cell invasion. Organotypic matrices were treated with vehicle or FAKi during contraction only (early stromal FAKi priming), during invasion only (late epithelial treatment), or during both contraction and invasion in a chronic treatment setting (Fig. 3L). Late epithelial treatment alone slightly decreased cell invasion in a dose-dependent manner (compare column 1 to 2 and 3; Fig. 3, M and N), in line with our previous results for KPC cell migration on CDMs (Fig. 2, B and C), while priming of the stroma before cancer cell seeding significantly decreased KPC cell invasion into the matrix that was not improved upon in a chronic treatment setting (compare column 1 with 4 to 7; Fig. 3, M and N) and was independent of changes in cancer cell proliferation or apoptosis (fig. S3, N and O). This demonstrated that short-term stromal FAKi priming can efficiently impair cancer cell invasion, while reducing the need for chronic treatment regimens, which can often lead to side effects in the case of stromal targeting (*3*, *7*) and opens up previously unidentified opportunities for less off-target effects in combination therapy in PC (*3*, *7*, *12*, *16*, *18*, *26*, *52*, *53*). We therefore sought to examine whether short-term FAKi priming of the stroma could improve the outcome of standard-of-care chemotherapy in PC.

### FAKi priming improves response to gemcitabine/Abraxane

**Fig. 4.**
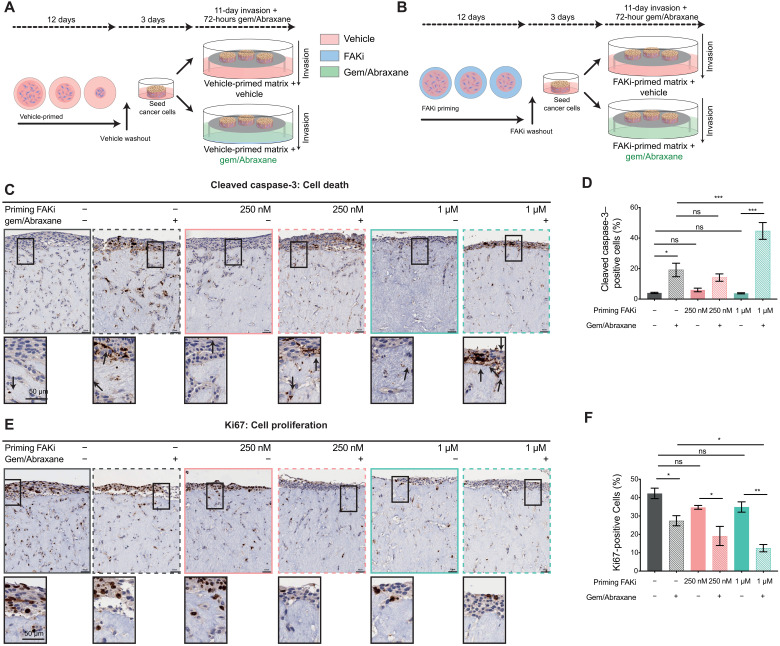
Manipulation of tumor-ECM feedback by FAKi priming improves KPC cell sensitivity to gemcitabine/Abraxane in organotypic matrices. (**A** and **B**) Schematic representation of an organotypic invasion assay where matrices were primed with vehicle (A) or FAKi (B) during contraction followed by FAKi washout, KPC cell seeding, 11-day invasion without drug treatment, and then 72 hours of gemcitabine (Gem) and Abraxane treatment. (**C** and **D**) Representative images of KPC cancer cells, stained for CC3 in vehicle or FAKi-primed matrices treated with control or gemcitabine/Abraxane (C) (scale bars, 50 μm; zoomed scale bar, 50 μm; arrows highlight positive cells) with quantification of CC3-positive cells invading into and on top of matrices (D). (**E** and **F**) Representative images of KPC cancer cells, stained for Ki67 in vehicle- or FAKi-primed matrices treated with control or gemcitabine/Abraxane (E) (scale bars, 50 μm; zoomed scale bar, 50 μm) with quantification of Ki67-positive cells invading into and on top of matrices (F). *n* = 3 biological repeats, 3 matrices per repeat, and 3 FOVs per matrix for quantification. Results: means ± SEM. *P* values were determined using an ordinary one-way ANOVA with Tukey correction for multiple comparisons. Unless otherwise indicated, significance is compared to vehicle. ns, *P* > 0.05; *P < 0.05, **P < 0.01, and ***P < 0.001.

Biomechanical input and ECM–cancer cell feedback have previously been shown to promote resistance to standard-of-care chemotherapies (*5*, *6*, *54*–*56*). As short-term stromal priming before invasion had a significant effect on the migratory capacity of KPC cells (Fig. 3, M and N) with the potential to minimize the need for chronic administration, we next investigated whether transient matrix manipulation by FAKi would sensitize KPC cells to subsequent standard-of-care gemcitabine/Abraxane in organotypic matrices (Fig. 4, A and B). CC3 and Ki67 staining in 3D-organotypic assays revealed that gemcitabine/Abraxane treatment alone increased KPC cell apoptosis and reduced cell proliferation in line with its clinical applications (Fig. 4, C to F, dark gray graphs versus hash gray graphs) (*2*). Stromal FAKi priming to impair fibroblast-mediated remodeling of the ECM, before gemcitabine/Abraxane treatment, however, resulted in a significant decrease in KPC cell proliferation and increase in KPC cell death compared to vehicle priming and gemcitabine/Abraxane treatment alone (Fig. 4, C to F, gray hash graphs versus green hash graphs). This effect was not improved upon with late FAK inhibition during KPC cell invasion (fig. S3, P to R, gray hash graph versus green hash graphs). In this way, short-term stromal targeting and matrix priming via FAK inhibition before chemotherapy may maximize current standard-of-care efficacy while minimizing potential side effects associated with long-term chronic stromal treatment. Guided by these data, we next assessed whether FAKi priming can improve response to chemotherapy in a live tumor setting.

### FAKi priming renders cells vulnerable to subsequent chemotherapy in live tumors

**Fig. 5.**
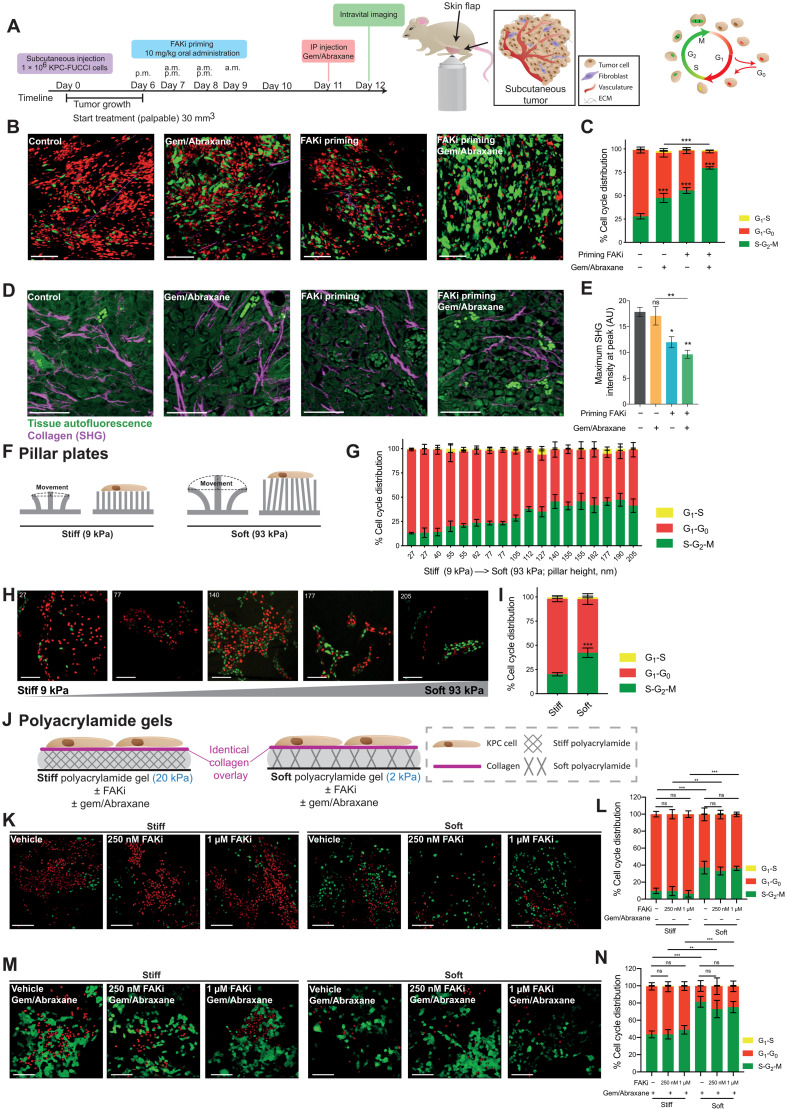
FAKi priming improves KPC tumor response to gemcitabine/Abraxane in vivo, which may partially be driven by a “softening” of the tumor microenvironment. (**A**) Timeline of subcutaneous KPC-FUCCI xenografts with intravital imaging of FUCCI cell cycle reporter in surgically exposed tumors. IP, intraperitoneal. (**B** and **C**) Representative FUCCI images (B) and quantification (C) in tumors primed with vehicle or FAKi followed by saline or gemcitabine/Abraxane. Scale bars, 50 μm. (**D** and **E**) Representative maximum projections of SHG signal (magenta) and tumor cells (green) (scale bars, 100 μm) (D) and quantification of peak SHG signal intensity (E). *n* = 5 animals per treatment group, 15 (B and C) and 5 FOVs (D and E) per animal. (**F**) Schematic of stiff and soft pillar plates. (**G** to **I**) Quantification (G), representative images (H) (scale bars, 50 μm), and pooled average cell cycle phase distribution (I) of FUCCI reporter in KPC-FUCCI cells grown on pillars of varying heights. Numbers indicate pillar height (increasing softness from left to right). Micropatterned cell surface interface differs for repeated pillar heights. *n* = 3 repeats, 1 height per micropattern per repeat, 5 FOVs per replicate. (**J**) Schematic of cancer cells seeded onto collagen-coated stiff (20 kPa) and soft (2 kPa) hydrogels. (**K** to **N**) Representative FUCCI images (K and M) (scale bars, 50 μm) and quantification (L and N) in KPC-FUCCI cells seeded onto hydrogels treated with vehicle or FAKi followed by saline (K and L) or gemcitabine/Abraxane (M and N). *n* = 3 biological repeats, 2 hydrogels per repeat per treatment group, 5 FOVs per gel. Results: means ± SEM. *P* values were determined using a two-way ANOVA (C, I, L, and N) and one-way ANOVA (E) with Tukey correction for multiple comparisons. ns, *P* > 0.05; *P < 0.05, **P < 0.01, and ***P < 0.001.

On the basis of our initial insights from 3D organotypic assays, we moved to a live intravital imaging setting to monitor in more detail the spatiotemporal response to FAKi priming in vivo. We generated FAK biosensor–expressing KPC cancer cells (KPC-FAK) and implanted them subcutaneously into mice. Once primary tumors were palpable, mice were primed with FAKi for 3 days before intravital imaging (see schematic in fig. S4A) (*18*, *33*). In vivo target validation using the FAK biosensor confirmed that short-term FAKi priming was sufficient to reduce FAK activity in live tumors (fig. S4, A to C, and movie S4) and also decreased ECM deposition and remodeling in the stromal compartment in vivo, as assessed by SHG imaging (fig. S4D). Using this priming approach, we next examined drug response in real time using the FUCCI cell cycle reporter in KPC cells (KPC-FUCCI) as a readout of chemotherapeutic efficiency, where cells change color (red to green) in real time depending on cell cycle stage (*31*, *34*). Following generation of palpable KPC-FUCCI tumors, mice were primed as described above, with FAKi for 3 days before gemcitabine/Abraxane treatment at day 11 (see schematic in Fig. 5A). Live tumor tissues were exposed 24 hours after chemotherapy and intravitally imaged (Fig. 5, A and B, and movie S5). Quantification of the FUCCI cell cycle reporter demonstrated that gemcitabine/Abraxane treatment alone resulted in a larger proportion of cells in S-G_2_-M phase compared to control [Fig. 5B (red-to-green shift, quantified in Fig. 5C)], as previously achieved (*31*) and in line with clinical applications. Priming with FAKi monotherapy revealed a similar increase in the proportion of cells in S-G_2_-M phase compared to control (Fig. 5, B and C), which may be due to direct effects of FAK inhibition on KPC tumor cells and indirect effects via changes in the tumor ECM architecture. Critically, priming with FAKi and subsequent chemotherapy induced a significantly larger proportion of cells in S-G_2_-M compared to gemcitabine/Abraxane alone, indicating an enhancement of chemotherapeutic efficiency (Fig. 5, B and C). IHC analysis of Ki67 and CC3 also confirmed that FAKi priming combined with gemcitabine/Abraxane reduced cancer cell proliferation and survival in vivo (fig. S4, E to H). To evaluate the direct or indirect effect of FAKi priming on KPC cells versus the tumor ECM architecture, we next aimed to dissect the epithelial and stromal effects of FAKi priming on KPC cell cycle distribution.

### Matrix stiffness rather than direct epithelial FAK inhibition dictates PC cell sensitivity to gemcitabine/Abraxane

SHG imaging along with polarized light microscopy of KPC tumors described above demonstrated that FAKi priming alone and before chemotherapy significantly reduced cross-linked fibrillar collagen deposition and organization (Fig. 5, D and E, and fig. S4, I to L). In line with the recognized role of outside-in signaling and ECM-tumor feedback in tumor progression (*14*, *44*, *57*), we next dissected the effects of epithelial and stromal FAK targeting on cell cycle progression using (i) the FUCCI biosensor, (ii) pillar plates (*58*, *59*), and (iii) stiffness-tunable matrices (*60*). The mechanoreciprocity between tumor cells and their surrounding ECM can influence several signaling pathways including those involved in tumor cell proliferation and cell cycle progression (*6*, *43*, *57*). Hence, the effect of matrix stiffness on cell cycle progression and chemotherapeutic efficiency was next assessed. Here, KPC-FUCCI cells were seeded onto polymethyl methacrylate pillar plates, where the pillar height (in nm) determines surface stiffness (*58*, *59*). Shorter pillars are less mobile and therefore have a stiffer surface, while taller pillars are more pliable and thus present a softer surface (Fig. 5F) (*58*, *59*). Here, 72 hours after seeding onto the pillar plates, KPC cells were imaged. Analysis of the FUCCI reporter demonstrated that the percentage of KPC cells in S-G_2_-M phase of the cell cycle increased in correlation with increasing pillar height and surface softness (Fig. 5, G and H). Grouping of the varying pillar heights into either stiff and soft (stiff, <112 nm and soft, ≥112 nm) revealed that there was a significant increase in the number of KPC cells in the S-G_2_-M phase on soft surfaces compared to stiff surfaces (Fig. 5I). This indicates that softer matrix surfaces can increase the number of KPC cells in S-G_2_-M, which may render them vulnerable to subsequent gemcitabine/Abraxane chemotherapy.

To further elucidate whether the observed increase in gemcitabine/Abraxane efficiency could be due to direct FAK inhibition or indirectly via changes in matrix ultrastructure and stiffness, KPC cells were seeded onto tunable soft or stiff polyacrylamide hydrogels (2 and 20 kPa, respectively) (*60*, *61*). Here, the area of direct cancer cell–matrix contact is equal, but the surface stiffness varies (see schematic in Fig. 5J). After seeding and adhesion to the hydrogels, cells were treated with vehicle or FAKi for 72 hours, followed by treatment with the vehicle control for gemcitabine/Abraxane for 24 hours. As before, cells grown on stiff versus soft gels revealed an accumulation of cells in S-G_2_-M in soft conditions (compare vehicle stiff and vehicle soft; Fig. 5, K and L). FAKi addition to cancer cells on these gels did not enhance this S-G_2_-M shift any further (compare FAKi stiff versus soft; Fig. 5, K and L). Next, cells were placed on stiff or soft hydrogels and subjected to gemcitabine/Abraxane for 24 hours after 72 hours of pretreatment and subsequent washout of vehicle or FAKi. Analysis of cell cycle distribution indicated by the FUCCI reporter revealed (i) that the shift to S-G_2_-M phase is again higher for cancer cells on soft gels than those on stiff surfaces, (ii) that gemcitabine/Abraxane increases the baseline number of cells in S-G_2_-M phase for all conditions (stiff and soft; compare Fig. 5, K and L, with Fig. 5, M and N), and (iii) that FAKi does not enhance the amount of cells in S-G_2_-M phase any further on both stiff and soft matrices in the presence of gemcitabine/Abraxane (Fig. 5, M and N). Together, this suggests that reducing matrix stiffness, rather than direct epithelial FAK inhibition, may increase KPC cell sensitivity to gemcitabine/Abraxane indirectly via stromal targeting and disruption of the ECM architecture, altering outside-in signaling and thereby increasing the number of KPC cells in S-G_2_-M, which may improve response to subsequent chemotherapy.

### Priming with FAKi reduces metastatic spread and sensitizes cells to chemotherapy during transit via enhanced vulnerability to shear stress and sensitivity to anchorage-independent growth

**Fig. 6.**
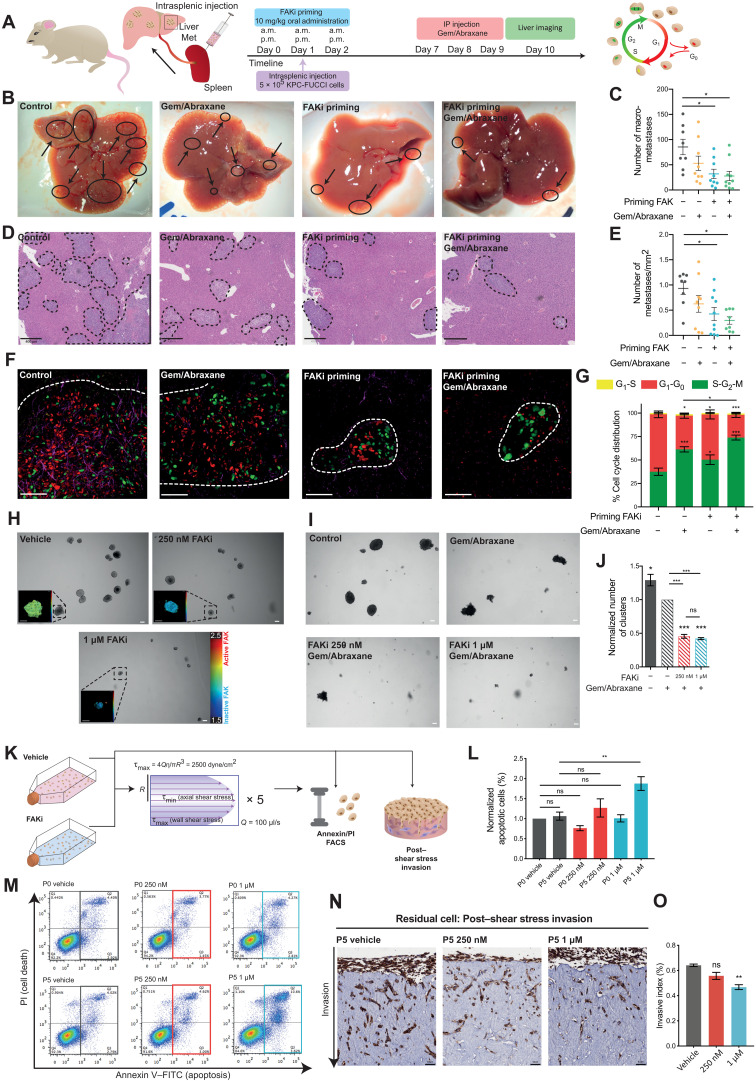
FAKi priming reduces metastasis and improves response to gemcitabine/Abraxane at secondary sites. (**A**) Timeline of KPC-FUCCI intrasplenic study. (**B** and **C**) Representative images (B) and quantification of visible liver metastases (C) upon priming with vehicle or FAKi followed by saline or gemcitabine/Abraxane. (**D** and **E**) Representative hematoxylin and eosin images (D) and quantification of liver metastases per square millimeter (E) (dotted lines). (**F** and **G**) Representative images (F) and quantification of FUCCI reporter (G) in KPC-FUCCI liver metastases. Scale bars, 50 μm. *n* = 8 (vehicle/saline), *n* = 9 (vehicle/gemcitabine/Abraxane), *n* = 9 (FAKi/saline), and *n* = 10 animals (FAKi/gemcitabine/Abraxane), ≥6 FOVs per animal (G). (**H**) Representative images of day 7 KPC AIG clusters treated with vehicle or FAKi (scale bars, 100 μm) with zoomed maps of ECFP fluorescence lifetime (scale bars, 50 μm). (**I** and **J**) Representative images (I) and quantification of KPC AIG cluster number (J) (normalized to vehicle/gemcitabine/Abraxane) primed with vehicle or FAKi followed by saline or gemcitabine/Abraxane. Scale bars, 100 μm. (**K**) Schematic representation of shear stress assay with KPC cells. (**L** and **M**) Quantification (L) and representative fluorescence-activated cell sorting (FACS) plots [annexin V/propidium iodide (PI)] (M) upon treatment with vehicle or FAKi and no shear stress (P0) or with shear stress (P5). (**N** and **O**) Representative images (N) and quantification of KPC invasion (O) treated with vehicle or FAKi before shear stress. Scale bars, 50 μm. *n* = 3 (AIG and invasion) and *n* = 5 (FACS) biological repeats, 3 replicates per treatment group per repeat. Results: means ± SEM. *P* values were determined using a two-way ANOVA (G) and one-way ANOVA (C, E, J, L, and O) with Tukey correction for multiple comparisons. Unless otherwise stated, significance is compared to vehicle. ns, *P* > 0.05; *P < 0.05, **P < 0.01, and ***P < 0.001. FITC, fluorescein isothiocyanate.

As the majority of patients with PDAC present clinically with locally invasive disease, we next investigated the potential of FAKi priming with chemotherapy to alter metastatic colonization at secondary sites in the liver or during transit (*18*, *62*, *63*). To mimic the effects of FAKi priming in the presence of circulating tumor cells, intrasplenic injections were performed using KPC-FUCCI cells in parallel with FAKi priming (Fig. 6A). Mice were primed with FAKi for 3 days during intrasplenic injection of tumor cells to mimic systemic or adjuvant therapy, and liver colonization was quantified (*18*, *31*, *63*). Quantification of visible macrometastases and histopathological analysis of serial sections through the liver showed that mice primed with FAKi alone or before gemcitabine/Abraxane, but not chemotherapy alone, had significantly fewer liver metastases than control conditions [Fig. 6B (macrometastases, quantified in Fig. 6C and confirmed in serial sections in Fig. 6, D and E)]. Furthermore, in line with our finding in the primary tumor (Fig. 5, B and C), live FUCCI imaging in the liver revealed that in control versus gemcitabine/Abraxane conditions, any metastases that were formed were evident with a clear shift to S-G_2_-M phase of the cell cycle in the chemotherapy arm [Fig. 6, F and G (compare panel 1 versus 2, red-to-green shift), and movie S6]. In the FAKi-primed condition, however, both the size and number of metastases were reduced, and in those small metastases that remained, there was also a significant shift of cells to S-G_2_-M for FAKi priming alone and combination therapy at secondary sites for residual metastases [Fig. 6, F and G (compare panel 3 versus 4, red-to-green shift), and movie S6]. This therefore reveals a global or systemic benefit of FAKi priming in the adjuvant disease setting in addition to its effect at the locally invasive primary site. Furthermore, CD31 staining in liver metastases showed that FAKi priming alone or in combination with chemotherapy resulted in smaller, less mature vasculature within metastases (fig. S5, A and B), indicating that FAKi priming may also disrupt the formation of nascent vessels at secondary sites or indirectly affect metastatic establishment due to reduced vascularization, in line with the known role of FAK in angiogenesis (*64*, *65*).

The reduced number of metastases observed here also led us to assess whether KPC cell survival in transit was affected by FAKi priming. We therefore initially exposed cancer cells to anchorage-independent growth (AIG; fig. S5C), where single KPC cells were suspended and grown in agarose to mimic anoikis during vascular transit, as previously achieved (*18*). Target validation using FLIM-FRET imaging of the FAK biosensor in these suspended cells revealed that FAK is also active in this 3D-detached setting, indicating that KPC cells could also be vulnerable to FAK inhibition in this context during transit [Fig. 6H (inset, showing FAK activity and response to FAKi treatment via FLIM-FRET imaging in suspension)]. Here, cells were initially treated with FAKi or vehicle over a 7-day period. Quantification of clusters revealed a significant decrease in clusters upon treatment with FAKi (fig. S5, C to E). We next exposed KPC cells treated with FAKi or vehicle in an AIG setting to subsequent gemcitabine/Abraxane for 72 hours (fig. S5F). Here, chemotherapy alone significantly reduced the number of clusters, which was further improved upon by priming with FAKi, again demonstrating a potential systemic advantage to FAKi priming in this highly aggressive and metastatic disease (Fig. 6, I and J).

In line with assessing FAKi priming under AIG conditions, we next used a shear stress model to mirror the physical forces and environmental stress that cancer cells may encounter while transiting in the vasculature (*18*, *66*). To mimic flow-induced shear stress forces found in the vasculature, cells were subjected to shear stress at a rate of 100 μl/s with a τ_max_ of 2500 dyne/cm^2^, for 5 cycles, following pretreatment with FAKi or vehicle, as previously achieved (*18*, *66*). After exposure to shear stress, cells were analyzed by flow cytometry for alterations in apoptosis via annexin V/propidium iodide (PI) staining and subjected to parallel testing of their post–shear stress invasive capacity (Fig. 6K, flow chart). Critically, no significant difference in apoptosis was observed in cells pretreated with FAKi before shear stress (P0); however, in cells that had been exposed to shear stress (P5), we observed a significant increase in apoptosis under FAKi-primed conditions (Fig. 6, L and M). In parallel, cell invasion for 14 days after exposure to shear stress was also examined and revealed a further inhibition of invasion under FAKi-primed conditions (Fig. 6, N and O). This indicates that beyond inhibiting primary PC invasion (Fig. 3, M and N), FAKi priming also has the capacity to affect tumor cell survival during transit in the circulation, and in addition, for those cells that do survive shear stress in the circulation, their capacity to invade and colonize secondary tissue may also be impaired.

### Patient-derived models reveal a potential to stratify patients for FAKi priming before chemotherapy in PDAC

**Fig. 7.**
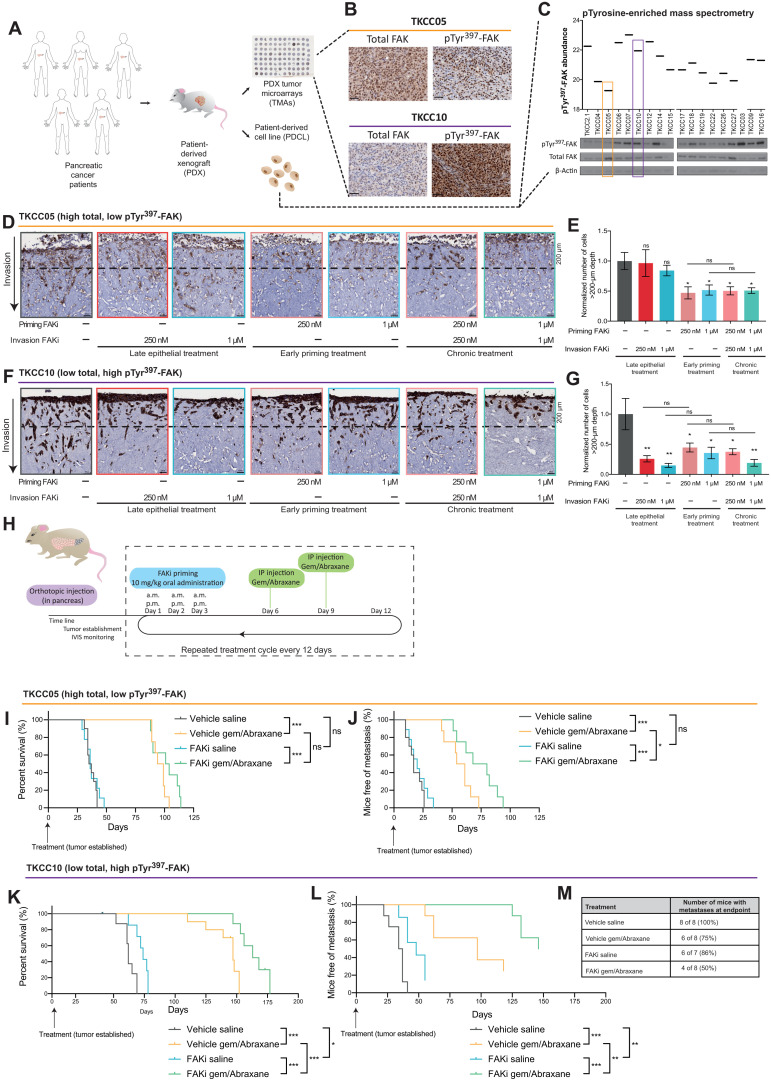
A FAK signature reveals a graded response to stromal and epithelial FAKi priming before gemcitabine/Abraxane. (**A**) Schematic representation of PDAC PDX, PDCL, and PDX TMA establishment. (**B**) Representative images of TKCC05 and TKCC10 PDX TMA samples stained with total and pTyr^397^-FAK. Scale bars, 50 μm; *n* = 3 cores per PDX. (**C**) Quantification of pTyr^397^-FAK levels in PDCLs [mass spectrometry, top; data obtained from (*67*)] and confirmation via Western blot (bottom), highlighting TKCC05 (orange; low active, high total FAK) and TKCC10 (purple; high active, low total FAK) cells. (**D** to **G**) Representative images (D and F) and quantification (E and G) (normalized cell number at >200-μm depth) of green fluorescent protein (GFP)–stained TKCC05 cells (D and E) and pan-cytokeratin–stained TKCC10 cells (F and G) invading into organotypic matrices primed with vehicle or FAKi (dotted line, 200-μm invasion depth). Scale bars, 50 μm. *n* = 3 biological repeats, 3 matrices per repeat, 3 FOVs per matrix. Results: means ± SEM. *P* values were determined using an ordinary one-way ANOVA with Tukey correction for multiple comparisons. Unless otherwise stated, all significance is compared to vehicle. (**H**) Timeline for TKCC05 and TKCC10 orthotopic studies. IVIS, In Vivo Imaging System. (**I** to **L**) Kaplan-Meier analysis of survival (I and K) and time to metastasis (J and L) in mice with TKCC05 (I and J) or TKCC10 (K and L) orthotopic tumors treated with vehicle/saline (TKCC05, *n* = 10; TKCC10, *n* = 8), vehicle/gemcitabine/Abraxane (TKCC05, *n* = 9; TKCC10, *n* = 8), FAKi/saline (TKCC05, *n* = 8; TKCC10, *n* = 7), or FAKi/gemcitabine/Abraxane (TKCC05, *n* = 8; TKCC10, *n* = 8 animals). (**M**) Percentage of TKCC10 mice with visible liver metastases at endpoint. Kaplan-Meier curves were compared using log-rank Mantel-Cox test. ns, *P* > 0.05; *P < 0.05, **P < 0.01, and ***P < 0.001.

PDAC has a highly heterogeneous molecular fingerprint, and intertumoral variability is a known hurdle in the lagging improvement of current therapies and patient survival. IHC staining for both active (pTyr^397^-FAK) and total FAK expression was performed on TMAs of patient-derived xenograft (PDX) tumors generated from the APGI cohort (Fig. 7, A and B) (*18*, *30*–*32*). Grading for staining intensity and coverage (fig. S1C), along with analysis of PDCLs by phosphotyrosine-enriched mass spectrometry and Western blotting (Fig. 7C) (*67*), identified heterogeneous FAK activity and expression across the PDCL panel. From here, we identified TKCC10 as an example of “high–FAK activity, low–FAK expression” signature and TKCC05 as an example of “low FAK activity, high FAK expression” [Fig. 7, B and C (orange versus purple box)]. These FAK activity and expression status are maintained upon subsequent orthotopic injection of isolated PDCLs into the pancreas [compare fig. S6, A and B (PDX tumors), with fig. S6, C and D (matched PDCL tumors), respectively]. Expression and FLIM-FRET imaging of the FAK-FRET biosensor in both TKCC05 and TKCC10 cells confirmed that both PDCLs maintain their FAK activation status with TKCC05-FAK cells exhibiting low lifetimes, indicating a low basal level of FAK activity, while TKCC10-FAK cells showed a high lifetime, indicating high FAK activity (fig. S6E).

To evaluate the potential of transient stromal manipulation and epithelial treatment on a personalized basis, TKCC05 and TKCC10 cells were seeded onto contracted organotypic matrices as previously performed using KPC cells (Fig. 3, A to C). Analysis of personalized organotypic matrices revealed that while low–FAK activity, high FAK–expression TKCC05 patient-derived cells did not respond to late epithelial treatment, FAKi priming and disruption of the matrix significantly decreased TKCC05 invasion into the matrix, which was not improved upon by chronic treatment (Fig. 7, D and E). This was independent of changes in survival or cell proliferation of cancer cells within the matrices (fig. S6, F and G). Invasion in the high–FAK activity, low–FAK expression TKCC10 patient-derived cells was significantly reduced upon both epithelial (late) and stromal (early) FAK inhibition, which was also not improved upon in a chronic treatment setting (Fig. 7, F and G). Again, this effect was independent of changes in cancer cell survival or cell proliferation in this setting (fig. S6, H and I). These data suggest that maximal benefits of FAKi priming may be evident where both epithelial and stromal FAK activities are present, while others may only benefit from stromal intervention.

Hence, we sought to assess the long-term effect of FAKi priming on therapeutic response to gemcitabine/Abraxane, metastasis, and survival in orthotopic settings by injecting luciferase-expressing TKCC05 or TKCC10 cells into the pancreas of mice. Following visualization of established primary tumors by whole-body In Vivo Imaging System (IVIS) imaging of the luciferase signal (average flux of 5.5 × 10^8^), mice were subjected to repeated cycles of FAKi priming for 3 days before gemcitabine/Abraxane treatment (see schematic in Fig. 7H). In the low–FAK activity, high–FAK expression TKCC05 model, survival was improved upon treatment with gemcitabine/Abraxane alone (Fig. 7I), and in line with our personalized organotypic invasion data (Fig. 7, D and E), time to metastasis was also increased in this setting (Fig. 7J). This reduction in metastatic progression, however, was not sufficient to significantly improve survival upon priming with FAKi alone or before chemotherapy (Fig. 7I and fig. S7). Conversely, treatment of high–FAK activity, low–FAK expression TKCC10 tumors with FAKi priming alone significantly improved survival compared to control [Fig. 7K and fig. S8; vehicle, saline (gray) mean survival: 62 days; FAKi, saline (blue) mean survival: 74 days]. Furthermore, FAKi priming before gemcitabine/Abraxane treatment also significantly improved survival compared to chemotherapy alone [Fig. 7K; vehicle, gemcitabine/Abraxane (yellow) mean survival: 147 days; FAKi priming, gemcitabine/Abraxane (green) mean survival: 163 days; fig. S8]. Time to metastasis was also significantly enhanced in this setting (Fig. 7L), and critically, in the FAKi combination arm with gemcitabine/Abraxane, the number of mice with macrometastases in the liver at endpoint was lowest (reduced to 50%; Fig. 7M) in addition to the significant survival advantage observed in those animals. Together, this graded response to FAKi priming before gemcitabine/Abraxane between TKCC05 and TKCC10 PDCLs highlights the need to identify valid biomarkers to guide combination treatment regimens in a personalized medicine approach to this highly heterogeneous disease, where the FAK activity status of patient tumors could be potentially used to identify patients who may benefit from FAKi priming before chemotherapy and recognize patients who may not respond to current clinical FAK-based targeted therapy in PC (*19*). In this way, our intravital assessment to dissect the benefits of FAK inhibition in PC could help guide future and ongoing clinical trials in this active arena of personalized medicine.

### Analysis of reduced Merlin expression as a potential biomarker for FAKi sensitivity in PC

**Fig. 8.**
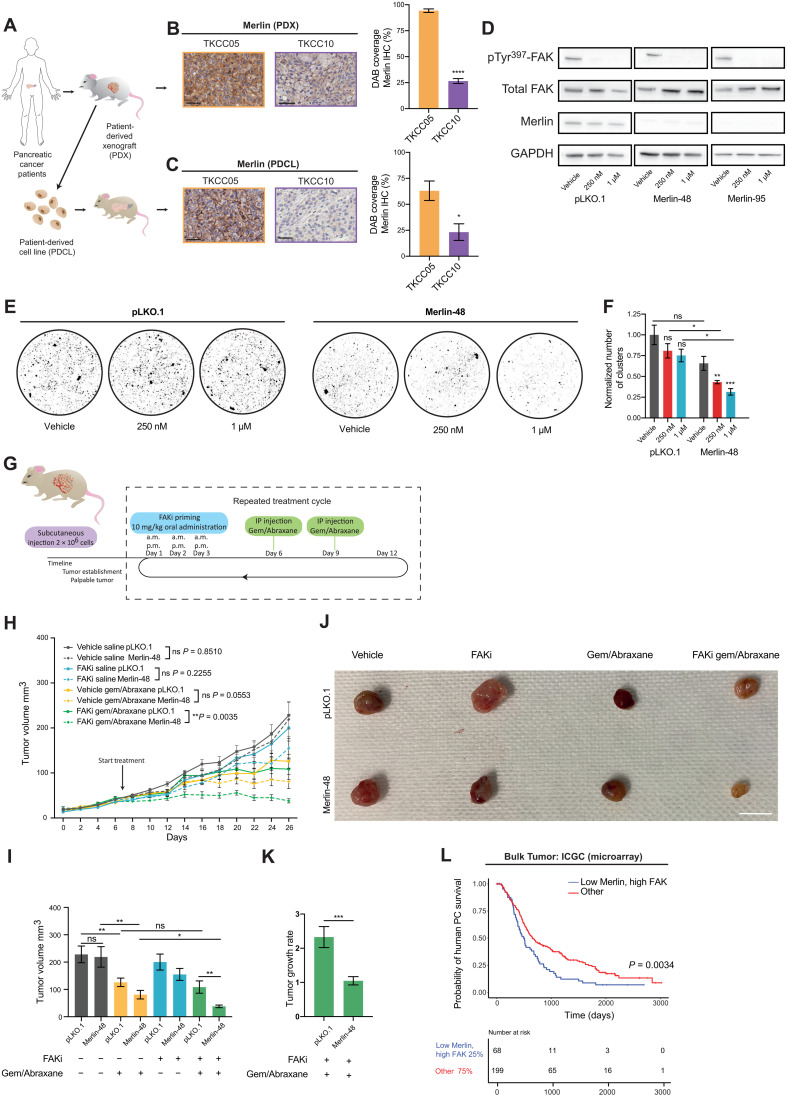
Manipulation of Merlin can sensitize nonresponsive patient-derived tumors to FAKi before chemotherapy and induce disease stabilization. (**A**) Schematics of establishment of PDXs, PDCLs, and PDCL-generated orthotopic tumors. (**B** and **C**) Representative Merlin-stained images and quantification of TKCC05 (orange) and TKCC10 (purple) PDX (B) and PDCL-generated tumors (C). Scale bars, 50 μm. (**D**) Western blot of pTyr^397^-FAK, total FAK, and Merlin in TKCC05 pLKO.1, Merlin-48, and Merlin-95 cells on CDMs treated with vehicle or FAKi. *n* = 3 repeats, 1 CDM per condition per repeat [quantified in fig. S9 (A to C.)]. GAPDH, glyceraldehyde-3-phosphate dehydrogenase. (**E** and **F**) Representative images (E) and quantification of TKCC05 pLKO.1 and Merlin-48 AIG assays (F) treated with vehicle or FAKi. *n* = 3 repeats, 4 replicates per treatment group per repeat. (**G**) Timeline for TKCC05 pLKO.1 and Merlin-48 tumors. (**H** and **I**) Quantification of TKCC05 pLKO.1 (solid) and Merlin-48 (dashed lines) tumor growth over time (H) and tumor size at day 26 (I) upon treatment with vehicle/saline (pLKO.1, *n* = 9; Merlin-48, *n* = 9), vehicle/gemcitabine/Abraxane (pLKO.1, *n* = 10; Merlin-48, *n* = 9), FAKi/saline (pLKO.1, *n* = 9; Merlin-48, *n* = 10), and FAKi/gemcitabine/Abraxane (pLKO.1, *n* = 8; Merlin-48, *n* = 10 animals). (**J**) Representative images of endpoint tumors. Scale bar, 1 cm. (**K**) Quantification of TKCC05 pLKO.1 and Merlin-48 tumor growth rate upon treatment with FAKi/gemcitabine/Abraxane. Results: means ± SEM. (**L**) Kaplan-Meier analysis of human PC survival correlating to low Merlin and high FAK versus others. *P* values were determined using unpaired two-tailed *t* test with Welsh correction for unequal variance (B, C, and K), one-way ANOVA with Tukey correction for multiple comparisons (F, H, and I), or log-rank test (L). Unless otherwise stated, all significance is compared to vehicle. ns, *P* > 0.05; *P < 0.05, **P < 0.01, and ***P < 0.001.

We next sought to establish a potential biomarker to stratify patients with PC for FAKi priming before chemotherapy. Merlin, the product of the neurofibromatosis type 2 (NF2) tumor suppressor gene, functions as a linker between transmembrane proteins and the actin cytoskeleton (*22*). It has recently been identified that in Merlin-high cells, stable cell-cell interactions decrease cell dependence on cell-ECM signaling through FAK (*20*, *22*). Conversely, Merlin-low cells rely on cell-ECM contacts, via integrin/FAK signaling, for their survival and proliferation, thus rendering them increasingly sensitive to FAK inhibition (*20*, *22*). Given the graded response observed between the two different patient tumors from in vitro and in vivo settings, tumors from both PDX models and orthotopic injections of PDCLs were stained for Merlin. This revealed that Merlin expression in original TKCC10 PDX tumors was significantly lower than in TKCC05 tumors (Fig. 8, A and B), which was maintained when PDCLs generated from these PDXs were orthotopically reimplanted to establish palpable tumors in the pancreas (Fig. 8C). Together, this suggests that the observed differences in sensitivity to FAK inhibition between the pancreatic PDCLs may be associated with different levels of Merlin expression, where low Merlin expression may be linked to improved response to FAKi, in line with preclinical and clinical studies in other cancers (*20*, *21*).

To investigate whether there is a causal relationship in PDAC between Merlin expression and cell response to FAKi, lentiviral short hairpin RNA (shRNA) targeting was used to reduce Merlin expression in the Merlin-high TKCC05 PDCL, which poorly responded to FAKi. We assessed stable pools of scrambled control and Merlin knockdown cells (henceforth pLKO.1, Merlin-48, and Merlin-95) to confirm Merlin knockdown (Fig. 8D and quantified in fig. S9A) and also show that Merlin knockdown did not alter FAK expression or activity [Fig. 8D and quantified in fig. S9 (B and C)]. Using AIG assays, where cells rely on cell-cell contact, we next assessed whether Merlin knockdown would sensitize cells in this setting to FAKi. A reduction in cell clusters in response to epithelial FAK inhibition was observed for Merlin-48 and Merlin-95 cells compared to pLKO.1 cells (Fig. 8, E and F, and fig. S9D), indicating a potentially improved response to FAKi in Merlin-low patient PDAC cells. We therefore examined in vivo whether decreased Merlin expression now renders TKCC05 PDCLs more sensitive to FAKi in combination with gemcitabine/Abraxane. Following establishment of palpable tumors (see schematic in Fig. 8G), mice were cycled through treatment rounds of FAKi for 3 days before gemcitabine/Abraxane treatment, as previously achieved (Fig. 7H). While we observed no significant difference in subsequent tumor growth between control and Merlin knockdown tumors for vehicle and single-agent treatments, we now observed a significantly improved response to FAKi and gemcitabine/Abraxane combination treatment in Merlin knockdown TKCC05 (Merlin-48) tumors compared to control (pLKO.1; Fig. 8, H to J, compare solid green line to dashed green line). This significantly enhanced response to FAKi priming and chemotherapy in Merlin knockdown tumors was also evident in representative tumors obtained 24 hours after the final chemotherapy cycle (Fig. 8J). Critically, in the Merlin knockdown setting, TKCC05 tumors failed to grow beyond the initial palpable stage upon combination treatment, resulting in stabilized disease [Fig. 8H, arrow, compare tumor volume between days 8 and 26 for dashed green line; Fig. 8K, pLKO.1 doubled in size (growth rate, 2.33), whereas Merlin-48 displayed stable disease (growth rate, 1.04)]. Last, these results are in line with analysis from patient data from the ICGC cohort (267 samples; Fig. 8L) in which low Merlin/high FAK significantly co-correlate with low survival in patients with PDAC and may represent a subset of patients with poor outcome/prognosis that could substantially benefit from FAKi priming before chemotherapy (Fig. 8L).

## DISCUSSION

Tumor-stroma cross-talk and mechanoreciprocity play a pivotal role in driving tumor establishment, growth, and metastasis (*14*, *16*, *44*). The characteristically dense desmoplastic stroma of PDAC is thought to promote tumor progression while hampering chemotherapeutic efficiency (*3*, *63*). Despite this, recent studies on stromal targeting have yielded conflicting results where total stroma ablation was seen to enhance tumor growth (*3*, *7*, *52*). However, alternative studies have indicated that transient manipulation of the matrix may be a promising strategy to reduce aggressive disease while minimizing the potential caveats of chronic stromal targeting (*9*, *11*, *15*, *18*, *26*). The integrin-FAK signaling axis transmits signals bidirectionally, permitting cells to sense and respond to their adjacent extracellular environment, which can become essential for cancer progression when cancer cells have to rely on interactions with their surrounding stroma [see schematic in fig. S9 (E to G)] (*12*–*14*, *22*). This study focused on short-term stromal and epithelial FAK inhibition, by (i) deconstructing transient stromal manipulation, which reduces inherent outside-in signaling and can render cells vulnerable to chemotherapy, while also (ii) evaluating direct targeting of epithelial FAK activity to interrupt intrinsic inside-out communication (*14*). Hence, streamlining short-term stromal and epithelial FAK targeting is shown to enhance chemotherapeutic efficiency, simultaneously having potential to reduce off-target effects often associated with long-term chronic combinatory treatment regimens, which may lead to undesirable drug interactions. For example, FAKi has previously been shown to inhibit the cytochrome P450 isoform CYP3A, which is the main metabolizing enzyme for taxane-based chemotherapeutics, such as Abraxane (*37*, *68*). Simultaneous FAKi and Abraxane administration can therefore potentially lead to poor drug clearance and toxicity, warranting the temporal separation and sequential administration of these treatments.

A combination of 2D and 3D in vitro techniques was used to deconstruct the benefits by which stromal and epithelial FAK inhibition may reduce PDAC aggressiveness in the context of gemcitabine/Abraxane chemotherapy. These studies may help guide which patient subgroup in this highly heterogeneous disease is likely to respond best to FAKi priming in combination with chemotherapy. Similarly, interpatient variability and/or intratumoral heterogeneity and response from this study, including Merlin assessment before treatment, warrant future assessment for combination with gemcitabine/Abraxane [see schematic in fig. S9 (E to H)] and other clinically relevant chemotherapy treatments. This may also include other combination applications in PC, such as the recent combination of FAKi with immunotherapy or mitogen-activated protein kinase kinase (MEK) inhibition in PC in which administration of multiple drug combinations and chronic treatment can serve as a bottleneck or a limitation for patient treatment in this disease (*8*, *15*, *53*, *69*, *70*). Moreover, our work also reveals that PDAC is susceptible to FAK inhibition during tumor transit and that FAKi priming has additional roles in enhancing PDAC apoptosis in response to shear stress and reducing subsequent post–shear stress invasion and colonization capacity in this setting (*62*, *66*). This is in line with the clinical management of PC since a large proportion of patients present with locally invasive and metastatic disease, indicating applicability of our study to adjuvant therapy in aggressive and invasive PC cases.

Resistance to chemotherapy is often described as a result of dysregulated membrane transporters and metabolic enzymes in the epithelial compartment, but stromal-tumor feedback and mechanoreciprocity between cells and their environment have also emerged as a key player in PC response to therapy (*18*, *43*, *52*, *54*, *56*, *71*). Our results suggest that the biomechanics of the environment rather than direct or chronic FAK inhibition can lead to a shift in PDAC cell cycle distribution that may favor chemotherapeutic performance. Future studies may provide further insight into how biomechanics regulate chemosensitivity, for example, by causing an accumulation of cells in S-G_2_-M, increasing the time that cells spend in S-G_2_-M, and/or inducing an initial S-G_2_-M cell cycle arrest, which could all make cells vulnerable for subsequent S-G_2_-M–targeting chemotherapy (*43*, *72*, *73*). ECM proteins, such as laminin, collagen I, and fibronectin have previously been associated with intrinsic resistance to chemotherapy in multiple cancers, including PC (*5*, *56*). Stromal targeting may reduce ECM content and biomechanics via blocking the de novo synthesis or cross-linking of ECM molecules or by increasing the production of ECM-degrading enzymes. We and others have previously shown that the Src/FAK signaling axis can regulate the secretion and activation of matrix metalloproteinases, which are known to proteolytically degrade ECM (*29*, *74*, *75*). This, in addition to reducing ongoing collagen cross-linking, could collectively contribute to the ECM reduction that we observe in vivo. Furthermore, recent work has demonstrated that targeting fibroblast contractility via Rho associated coiled-coil containing protein kinase (ROCK) inhibition significantly reduces cancer progression (*17*), and in particular, transient stromal priming via ROCK inhibition reduces ECM organization and stiffness and sensitizes PDAC cells to chemotherapy while also reducing their metastatic capacity (*18*). This is in line with similar studies whereby inhibition of collagen cross-linking through Lysyl oxidase (LOX) inhibition in combination with gemcitabine impaired PDAC progression (*6*). In addition, reprogramming of the PDAC tumor stroma by long-term FAK inhibition leads to the acquisition of therapeutic resistance (*53*), suggesting that in a chronic treatment setting, resistance to stromal targeted therapies can emerge. These studies highlight the importance of the ECM in response to standard-of-care chemotherapy and provide a rationale for how short-term priming rather than chronic stromal treatment may be essential to maintain treatment durability and enhance gemcitabine/Abraxane therapy. Notably, assessing surgical human PDAC samples from the APMA cohort, which have been treated presurgically with chemotherapy, readily demonstrated an increase in fibrosis upon neoadjuvant treatment, suggesting that stromal priming before treatment could also have clinical benefits in blunting this fibrotic reaction in PDAC.

Personalized medicine is an emerging treatment option in the clinical management of PDAC and could potentially improve upon traditional “one-size-fits-all” approaches. Here, individual tumors are treated on the basis of a defined phenotype and the identification of predictive biomarkers of response to frequently used therapeutics (*3*, *20*). Personalized therapy has offered progresses in patient survival outcomes in a number of cancer types (*19*). This highlights the critical need to rapidly identify and validate prognostic and predictive biomarkers for drug response in PC. Here, using intravital imaging in live tumors, we identified a graded response to transient epithelial and stromal FAK inhibition in reducing PDAC invasiveness and also rendering a subtype of patients with PC, with high FAK and low Merlin signature, exquisitely vulnerable to subsequent chemotherapy. In line with previous observations in patients with mesothelioma (*20*, *21*), we reveal that Merlin status may also predict for FAKi sensitivity in PDAC, and on the basis of our preclinical data using patient-derived assessment, this may warrant further investigation. Molecular manipulation of Merlin in previously poorly responding TKCC05 PDCLs resulted in a significant shift in response in vivo and induced stabilized disease in this aggressive model. These results are in line with data from patient samples from the ICGC, in which low Merlin/high FAK status co-correlate with poor patient survival in PC and may represent a subset of patients who could substantially benefit from FAKi priming with chemotherapy in a stratified manner. The identification of potential biomarkers for treatment response, such as Merlin, may therefore facilitate which patients with PDAC are most likely to benefit from short-term FAKi priming before chemotherapy and warrants future assessment in this disease. Collectively this work, demonstrates that short-term FAK inhibition improves chemotherapeutic efficiency in a subset of patients with PDAC and may be used to guide ongoing clinical trials involving FAK targeting in this highly aggressive and metastatic cancer.

## MATERIALS AND METHODS

### Study design

This study assesses the potential of short-term transient manipulation of the stromal microenvironment via FAK inhibition before gemcitabine/Abraxane chemotherapy in PDAC. In vitro CDMs, organotypic matrices, and AIG assays were conducted in independent biological triplicates with three technical replicates per repeat and treatment group. Mouse numbers used in in vivo experiments are outlined in the corresponding figure legends. For in vivo priming studies, treatment began when tumors were palpable and had an average volume of 30 mm^3^ or average IVIS flux of 5.5 × 10^8^.

FLIM-FRET analysis of the FAK biosensor was conducted on 50 cells per group in three independent biological repeats for in vitro experiments and 100 cells per mouse for in vivo experiments. For FUCCI cell cycle analysis, in vitro experiments used at least three fields of view (FOVs). In vivo analysis was performed on 15 regions of interest (ROIs) for subcutaneous tumors and up to 15 metastases where possible for intrasplenic injections.

FAK and Merlin expression was assessed via the analysis of mRNA expression from the ICGC datasets. mRNA expression was segregated into quartiles, and Kaplan-Meier curves were generated with the log-rank test used to determine significance. PC arrays from the APGI cohort were used to assess stromal collagen levels and phosphorylated FAK (pTyr^397^) via IHC in a cohort of 231 patients (158 deceased with complete datasets) with untreated and operable primary tumors who had undergone pancreatectomy. In deceased patients with low ECM levels (Picrosirius coverage), pTyr^397^-FAK levels were segregated into high and low groups and Kaplan-Meier curves were generated.

Unless otherwise stated in the corresponding figure legends, all IHC, SHG, GLCM, and Picrosirius red analyses were conducted on three representative ROIs in organotypic matrices and five ROIs for CDMs, subcutaneous xenografts, and intrasplenic experiments. Liver metastatic burden following intrasplenic injection was analyzed on serial sections. Study endpoints for in vivo experiments were in compliance with the Garvan/St. Vincent’s Animal Ethics Committee (13/17, 14/06, 16/13, 19/10, and 19/13) and the Australian code of practice for care and use of animals for scientific purposes.

### Statistical analysis

Statistical analysis was performed using GraphPad Prism (GraphPad Software Inc., CA) with statistical significance given as not significant (ns) *P* > 0.05, *P < 0.05, **P < 0.01, and ***P < 0.001. For mRNA expression analysis of the ICGC cohort, statistical tests were performed using R (version 4.0.2). For data normalized to 1, a one-sample *t* test was performed. FUCCI cell cycle data analysis was assessed using a two-way analysis of variance (ANOVA) with Tukey correction for multiple comparisons. Kaplan-Meier curves were compared using a log-rank Mantel-Cox test. Unless otherwise stated, following normality conformation, all other data were analyzed using an ordinary one-way ANOVA with a Tukey correction for multiple comparisons.

### Animals

Animal experiments were conducted in accordance with the Garvan/St Vincent’s Animal Ethics Committee guidelines (13/17, 14/06, 16/13, 19/10, and 19/13) and in compliance with the Australian code of practice for care and use of animals for scientific purposes. Mice were kept in individual ventilated, isolated cages on a 12-hour light/12-hour dark cycle and fed ab libitum.

### Drug treatment schedules

Stock solutions of FAKi (PF-562271, SelleckChem) were prepared at 50 mM in dimethyl sulfoxide (DMSO) for in vitro utilization, while, for in vivo studies, FAKi was dissolved in DMSO and diluted in 20% (w/v) Gelucire 44/14 (Gattefosse, 3051) and sterile water to a final concentration of 5% FAKi/vehicle, 5% (w/v) Gelucire 44/14, and 90% sterile water. In vitro, FAKi was used at 250 nM and 1 μM concentrations in cell culture medium with DMSO as vehicle control. To assess chemotherapeutic response, gemcitabine (Jomar Life Research) and nab-paclitaxel (Abraxane, Specialised Therapeutics) were used at 100 nM each in tissue culture medium with saline as vehicle control.

In vivo, FAKi (PF-562271, SelleckChem) was administered twice daily by oral gavage for 3 days, using a 22-gauge feeding tube (Instech Laboratories, FTP-22-25) coated in 24% sucrose solution (Sigma-Aldrich, S9378). In subcutaneous models, treatment commenced 6 days after KPC cell injection when tumors had reached an average size of 30 mm^3^. For intrasplenic models, mice were subjected to three treatments with FAKi before and after intrasplenic injection of KPC cells. In pancreatic orthotopic models, FAKi treatment began when tumors were palpable and detectable by IVIS imaging (average flux of 5.5 × 10^8^) around 1 week after intrapancreatic injection for TKCC05 cells and around 4 weeks for TKCC10 cells. Nab-paclitaxel (30 mg/kg; Abraxane, Specialised Therapeutics) and gemcitabine (70 mg/kg; Jomar Life Research) were dissolved in sterile saline and administered by intraperitoneal injection in a treatment schedule, as outlined in the corresponding figures and figure legends.

### Cloning and generation of plasmids

The lipid raft/membrane–localized intramolecular Lyn-FAK FRET biosensor was provided by Y.W., University of California, Los Angeles (*33*). The Lyn-FAK biosensor was subcloned into the Gateway shuttle vector pENTR2b (Thermo Fisher Scientific, A10463) and amplified using competent *Escherichia coli* (DH5α, Thermo Fisher Scientific) cells. The pENTR2b-Lyn-FAK construct was then subcloned into the Gateway compatible PiggyBac transposon–based destination vector (pPBDEST51; Thermo Fisher Scientific, 12285011), by LR reaction using LR Clonase II as per the manufacturer’s instructions to generate pPBDEST-Lyn-FAK. All sequencing was performed in-house at the Garvan Molecular Genetics facility (Sydney, Australia) and verified using SnapGene (GSL Biotech: snapgene.com).

### Cell culture

Primary 83,320 KPC cells have previously been isolated from end-stage *Pdx1-Cre*, *LSL-Kras^G12D/+^*, *LSL-Trp53^R172H/+^* KPC mice (*27*, *29*, *35*). Telomerase-immortalized fibroblasts (TIFs) were also generated previously (*18*, *28*, *76*). KPC cells, TIFs, and human embryonic kidney (HEK) 239T cells were maintained in Dulbecco’s modified Eagle’s media (DMEM; high glucose, pyruvate; Gibco) supplemented with 10% fetal bovine serum (FBS; HyClone) and 10 mM Hepes (Gibco). TKCC05 cells were maintained in media consisting of DMEM/Ham’s F12 media (Gibco) supplemented with 7.5% FBS, 10 mM Hepes, 13.3 mM glucose, hydrocortisone (40 ng/ml), insulin (0.1 IU/ml), and human recombinant epidermal growth factor (EGF; 10 ng/ml). TKCC10 cells were maintained in 1:1 M199 media/Ham’s F12 medium (Gibco) supplemented with 7.5% FBS, 15 mM Hepes, 2 mM glutamine, 1× MEM vitamins, apotransferrin (25 ng/ml), insulin (0.2 IU/ml), 6.5 mM glucose, hydrocortisone (40 ng/ml), EGF (20 ng/ml), triiodothyronine (0.5 pg/ml), and *O*-phosphoryl ethanolamine (2 μg/ml). All cells were cultured in the presence of penicillin-streptomycin (100 U/ml and 100 mg/ml, respectively) and maintained at 37°C and 5% CO_2_. Experiments were conducted at 20% oxygen in a Heracell 150i CO_2_/O_2_ incubator for KPC, TIF, and TKCC05 cell lines and at 5% oxygen for TKCC10 lines. All cell lines were mycoplasma-free.

### Generation of stable cell lines

Generation of stable cell lines expressing the pPBDEST-Lyn-FAK biosensor was achieved by cotransfection of the pPBDEST-Lyn-FAK vector and pCMV-hyPBase (obtained from the Wellcome Trust Sanger Institute) (*77*) using Lipofectamine 3000 Reagent as per the manufacturer’s instructions. Positive cells were selected by fluorescence-activated cell sorting (FACS). Cancer cells were engineered to express the FUCCI cell cycle reporter (mKO2-hCdt1 and mAG-hGeminin) (*31*), Luciferase-GFP (green fluorescent protein; pLV430G), and pLKO.1 or Merlin targeting shRNA using a third generation lentiviral packaging system. Lentiviral particles were generated using HEK293T cells. Briefly, cells were treated with the transfection mixture containing pMDLg/pRRE (Addgene, plasmid 12251), pRSV-Rev (Addgene, plasmid 12253), pMD2.g (Addgene, plasmid 12259), plasmid DNA, and Lipofectamine 3000, as per the manufacturer’s protocol, and the medium was replenished after 24 hours. Lentiviral particles were harvested by filtration through a 45-μm filter. KPC, TKCC05, and TKCC10 cells were transduced with lentiviral dilutions for 48 hours in the presence of Polybrene (8 μg/ml) and washed, and stable positive cells were then selected using FACS (fluorescent constructs) or puromycin (2 μg/ml; p.LKO.1 and Merlin shRNA).

### AIG assays

AIG assays were performed as previously described (*18*). Briefly, 2 ml of cell-free 1% Low Melting Point Agarose (Quantum Scientific) was added to the base of six-well plates and allowed to solidify at room temperature for 1 hour. KPC cells were trypsinized, filtered (40-μm cell strainer), and diluted to obtain a single-cell suspension at 1000 cells/ml. Five hundred microliters of this cell suspension was then mixed with 1 ml of 0.3% agarose and plated over the solidified 1% agarose layer. The mixture was allowed to set at room temperature for 30 min and imaged to validate the absence of cell clusters. Cell clusters arising from single cells were allowed to grow in the presence of vehicle or FAKi for 7 days for monotherapy. For combination therapy, cells were treated with vehicle or FAKi until day 6, followed by the addition of 100 nM gemcitabine and 100 nM Abraxane for an additional 72 hours. Images for analysis of cluster size were acquired on a DM4000 Leica microscope, and the size was then measured on ImageJ (see the “AIG cluster size” section in “Macros”). To analyze total cell cluster number, assays were stained with Quick Dip and imaged using a Leica MZ12.5 microscope to obtain images of the entire well and quantified in ImageJ.

### CDM assays

CDMs were established as previously described using TIFs (*18*, *28*, *36*, *76*). To generate CDMs on glass, the surface was coated with 2% gelatin and allowed to set at 37°C for 2 hours then rinsed twice with Dulbecco’s phosphate-buffered saline (PBS) and formalin-fixed at room temperature for 30 min. After two rinses with PBS, fixed gelatin cross-links were quenched in 1 M sterile glycine for 30 min at room temperature, and coated plates were rinsed twice with PBS and once with DMEM before TIF cell seeding. TIFs were allowed to expand until confluent. Medium containing ascorbic acid (50 mg/ml) was then refreshed every other day for 7 days. For FAK inhibition, FAKi was added to the media at day 1 and refreshed on day 4. TIFs were removed at day 7 [extraction buffer: 0.5% (v/v) Triton X-100, 20 mM ammonium hydroxide, and 1% (w/v) sodium deoxycholate], and CDMs were rinsed with PBS before SHG imaging, seeding of cancer cells, or staining with Picrosirius red. After removal of the fibroblasts, 2 × 10^4^ KPC cells were seeded on the CDMs and allowed to adhere. For migration assays, KPC cells were allowed to adhere to the matrices for 8 hours before the addition of FAKi and subsequent live cell imaging for 8 hours. Tracking and quantification of cell migration were performed on binary images using a MATLAB plugin (CellTracker) (*78*). Representative tracks were plotted using MATLAB (MathWorks, USA). Cell streaming was monitored for 72 hours after KPC cell adhesion to the CDMs on an Incucyte. Images of matrix anisotropy and collective cell streaming were equally segmented into nine areas, and anisotropy (as a measure of coordinated migration) was quantified using the ImageJ plugin FibrilTool (*42*). Matrix fibrillar organization was analyzed using the ImageJ macro TWOMBLI (*46*) on CDMs with intact fibroblasts.

### Organotypic invasion assay

#### Collagen extraction

Collagen I was extracted from rat tails as previously described (*18*, *26*). Briefly, frozen rat tails were thawed, and collagen tendons were pulled free of the epithelium and skeletal structure using three-pronged tweezers. Extracted tendons from 10 to 12 tails were then solubilized in 1500 ml of 0.5 M acetic acid for 48 hours at 4°C on a magnetic stirrer. The mixture was then filtered through a strainer to remove sheath and insolubilized tendons before precipitation with 10% (w/v) sodium chloride on a magnetic stirrer over 6 to 8 hours. Once a homogenous opaque white solution was formed, the mixture was centrifuged at 10,000 rpm for 30 min at 4°C. The collagen precipitate was then dissolved in 400 to 600 ml of 0.25 M acetic acid overnight at 4°C on a magnetic stirrer, and the solution was then dialyzed in 4 liters of 17.4 mM acetic acid.

#### Contraction assays

Organotypic matrices were generated as described in (*18*, *26*). Briefly, 8 × 10^4^ TIFs per matrix were embedded in a sodium hydroxide–neutralized preparation of acid-extracted rat tail collagen (~2.5 mg/ml) in the presence of 1× MEM and 8.8% FBS. Matrices were allowed to set at 37°C before being detached and allowed to contract for 12 days in DMEM with 10% FBS, 10 mM Hepes, and penicillin-streptomycin (100 U/ml and 100 mg/ml, respectively). For early treatment (priming), matrices were treated during contraction with vehicle or 250 nM or 1 μM FAKi at day 0 and refreshed at day 6.

#### Invasion assays

After 12 days of contraction, TIF-contracted matrices were seeded with 1 × 10^5^ KPC cells, 1 × 10^5^ TKCC05 cells, or 1.5 × 10^5^ TKCC10 cells. After 72 hours of cancer cell growth, seeded matrices were moved to an air-liquid interface on a metal grid, and cancer cells were allowed to invade into the matrix for 14 days for KPC cells and for 21 days for TKCC05 and TKCC10 cells. For late treatment regimes, medium was supplemented with vehicle, 250 nM FAKi, or 1 μM FAKi and refreshed three times per week during cell invasion. Chronic treatment involved FAKi treatment during both matrix contraction and invasion. To assess chemotherapeutic response, 100 nM gemcitabine and 100 nM Abraxane were added for the final 72 hours of invasion. Matrices were then fixed in 10% neutral buffered formalin and processed for paraffin block embedding, sectioning, and histological and IHC analysis (Garvan Histopathology Facility). For KPC-FAK and KPC-FUCCI imaging of invasion into organotypic matrices, matrices were imaged before fixation. Using cell numbers calculated from pan-cytokeratin staining for KPC and TKCC10 cells and anti-GFP staining for TKCC05 cells, cell invasion index was calculated as the total number of cells invading greater than 200-μm depth divided by the cells on top of the matrix and normalized to the average invasion index of vehicle cells

$$\text{Invasive index}=\frac{\text{Number of cells}>200\;\mu m\;\text{depth}}{\text{Cells on top of the matrix}}$$

### Fluid shear stress assays

KPC cells were cultured in the presence of vehicle or FAKi (250 nM and 1 μM, refreshed every 24 hours) for 72 hours before being subjected to shear stress as previously described (*18*, *66*). Briefly, cells were trypsinized and filtered to generate a single-cell suspension and resuspended at a concentration of 5 × 10^5^ cells/ml in DMEM, and an aliquot designated as “P0” was isolated, which was not exposed to shear stress. The remaining cell suspension, “P5,” was exposed to five manual repeated passages of shear stress through a 30-gauge needle at a constant flow rate of 100 μl/s. Here, Poiseuille’s equation was used to measure shear stress, τ_max_ = 4*Q*η/π*R*^3^, whereby *Q* is the flow rate (0.1 cm^3^/s), η is the dynamic fluid viscosity of the cell culture medium at room temperature (0.78 × 10^−3^ N•s/m^2^), and *R* is the radius of the needle (*R* = 7.94 × 10^−3^ cm), resulting in τ_max_ = 2500 dyne/cm^2^ (*66*). After exposure to shear stress, 5 × 10^5^ cells were seeded into tissue culture flasks and used to assess cell death 24 hours after shear stress by flow cytometry using annexin V and PI staining. P0 and P5 cells (1 × 10^5^) were seeded onto TIF-contracted matrices and allowed to adhere for 4 days before being moved to an air-liquid interface for 14-day invasions with untreated media refreshed three times weekly.

### Fluorescence-activated cell sorting

FACS of cells was performed using the FACSCanto II (Becton Dickinson Biosciences) for annexin V/PI staining. For isolation of stable cell lines, a FACSAria III (Becton Dickinson Biosciences) was used. All quantifications were performed in FlowJo software (Tree Star Inc.).

Transfected cells were expanded and resuspended at 1 × 10^6^ cells/ml. For isolation of pbDEST-Lyn-FAK–transfected cells, yellow fluorescent protein–positive cells within a similar fluorescent range were selected. To obtain FUCCI-transfected stable cells, cells transfected with mKO2-Cdt1 (YG582/15-A) with a similar fluorescent range were selected. Following a further transfection with mAG-hGeminin (B530/30-A), again, cells with a similar fluorescent range were selected.

To analyze cell death following exposure to shear stress, an annexin V (fluorescein isothiocyanate)/PI staining kit was used on unfixed cells as per the manufacturer’s instructions. Quantification was performed in FlowJo software (Tree Star Inc.). A minimum of 50,000 events were analyzed per sample.

### ICGC, APGI, and APMA patient data

FAK and Merlin expression was assessed via the analysis of mRNA expression from the ICGC microarray datasets. mRNA expression was segregated into quartiles, and Kaplan-Meier curves were generated with the log-rank test used to determine significance. Statistical tests were performed using R version 4.0.2. For IHC analysis of patient TMAs from the APGI cohort, tumor cores of deceased patients (three per patient) with complete survival datasets (total, 158) were scored for stromal intensity (Picrosirius red) and segregated into low and high. Within the low–Picrosirius red cohort of patients, the stroma was then assessed for pTyr^397^-FAK intensity and further segregated into high and low. Kaplan-Meier curves were generated, and a log-rank test was performed to determine significance. For analysis of APMA patient samples, surgical samples from *n* = 3 treatment-naïve patients and *n* = 4 neoadjuvant gemcitabine/Abraxane-treated patients were analyzed for Picrosirius red coverage (*n* = 5 FOVs per tumor).

### Pillar plates

Multiwell array pillar plate slides were fabricated from poly(methyl methacrylate) as previously performed by the laboratory of N.G. (*58*, *59*). Pillar stiffness ranges from 9 kPa (height, 27 nm) to 93 kPa (height, 205 nm) (*58*, *59*). Individual pillars are 100 nm in diameter and spaced 300 nm apart and of varying height to obtain surfaces of different stiffness. KPC-FUCCI cells (2 × 10^3^) were seeded onto pillar plates and allowed to adhere and proliferate for 72 hours before being imaged using confocal microscopy.

### Polyacrylamide hydrogels

Polyacrylamide hydrogels with defined mechanical properties were prepared as previously described (*15*, *60*, *61*). Briefly, polyacrylamide solutions (3% acrylamide/0.1% bis-acrylamide for soft conditions and 6% acrylamide/0.1% bis-acrylamide for stiff conditions, with stiffness confirmed by bulk unconfined compression) were prepared in PBS and degassed for 5 min to remove all oxygen. Before gel formation, coverslips were functionalized, to permit robust hydrogel attachment, for 5 min in functionalization solution [3% acetic acid and 0.5% 3-(trimethoxysilyl)propyl methacrylate in absolute ethanol]. Glass slides were treated with dichlorodimethylsilane to create a hydrophobic surface and easy hydrogel detachment following the completion of gelation.

Hydrogel gelation kinetics were initiated by adding 0.1% (w/v) freshly made ammonium persulfate and 0.1% tetramethyl-ethylenediamine. Hydrogel solution (100 μl) was added to the glass side and sandwiched between the functionalized coverslip and hydrophobic glass slide. The gels were allowed to set for half an hour before careful removal from the dichlorodimethylsilane-treated glass slide. Bulk unconfined compression analysis was used to quantify the stiffness of hydrogels at 2 kPa (soft) and 20 kPa (stiff) (*15*). All gels were thoroughly washed with PBS overnight and stored at 4°C until further use. To enable cell attachment, gels were functionalized with rat tail collagen. For this, gels were washed three times with 50 mM Hepes buffer (pH 8.5), followed by incubation with sulfo-SANPAH (0.2 mg/ml) in 50 mM Hepes. The sulfo-SANPAH was then activated with ultraviolet (UV) light at 365 nm for 10 min using a UV cross-linker box. The gels were then washed twice for 5 min with 50 mM Hepes and incubated overnight at 4°C with rat tail collagen (0.08 mg/ml; diluted in 17.4 mM acetic acid). Last, after three 5-min washes with PBS, the gels were sterilized under UV light for 20 min before cell seeding. Here, 5 × 10^3^ KPC-FUCCI cells were seeded onto sterilized gels and allowed to adhere and proliferate for 24 hours before treatment with vehicle or FAKi (250 nM or 1 μM) for 48 hours, followed by washout and treatment with 100 nM gemcitabine and 100 nM Abraxane for 24 hours. KPC-FUCCI cells were then imaged by confocal microscopy.

### Scratch wound assay

For polarization assays, 1.5 × 10^5^ KPC cells were seeded onto glass coverslips and grown until confluency. Monolayers were then wounded with a pipette tip and washed twice with PBS. Wounds were allowed to close over 2 hours in the presence of vehicle or FAKi (250 nM or 1 μM) before fixation in 4% paraformaldehyde, and immunofluorescence staining of GM-130 was performed.

### Intrasplenic injections

KPC-FUCCI cells (5 × 10^5^ cells in 50 μl of PBS) were injected into the spleen of BALB/c-Fox1nuAusb mice [anesthetized with 3% isoflurane, O_2_ (1 liter/min), with continuous vacuum to remove excess isoflurane] as previously described (*18*). Briefly, a left subcostal incision was made through the skin and the peritoneum exposing the spleen, into which tumor cells were injected using a 29-gauge needle. The injection site on the spleen was then sealed with cyanoacrylate, and the peritoneal wall and skin were individually sutured while treating topically with analgesia (Bupivicaine). Mice were subjected to three treatments with FAKi (10 mg/kg) or vehicle by oral gavage before intrasplenic injection, followed by three subsequent treatments. Furthermore, mice were treated with gemcitabine (70 mg/kg) and Abraxane (30 mg/kg) or saline on days 7, 8, and 9 after injection, as previously performed (*18*). On day 10 mice, were euthanized for counting of visible metastasis and for imaging of liver metastases. Fresh organs were harvested and immediately imaged at 37°C on a heated stage (maximum duration of 1 hour after euthanasia). Imaging of the FUCCI cell cycle reporter was performed by multiphoton microscopy. Following imaging of liver metastases, livers were fixed in formalin for histological processing.

### Subcutaneous injections and intravital imaging

KPC-FAK cells, KPC-FUCCI cells, TKCC05 pLKO.1 cells, or TKCC05 Merlin-48 cells were injected into the rear flank of BALB/c-Fox1nuAusb mice while under anesthesia [3% isoflurane, O_2_ (1 liter/min), with continuous vacuum to remove excess isoflurane]. KPC cells were injected at 1 × 10^6^ in 50 μl of PBS, and TKCC05 cells were injected at 2 × 10^6^ in 80 μl of PBS/Matrigel (1:1). Tumors were allowed to develop for 6 days to an average volume of 30 mm^3^ for KPC tumors before treatment schedules commenced. For KPC-FAK–derived tumors, mice were treated with FAKi (10 mg/kg) or vehicle twice daily for 3 days with the final treatment 2 hours before skin flap surgery and subsequent intravital imaging. For KPC-FUCCI–derived tumors, mice were treated with FAKi (10 mg/kg) or vehicle twice daily for 3 days (6:00 p.m. and 7:00, 8:00, and 9:00 a.m.) before a single treatment with gemcitabine (70 mg/kg) and Abraxane (30 mg/kg) or saline on day 11, 24 hours before skin flap surgery and subsequent imaging. Mice were terminally anaesthetized using a mix of xylazine (10 mg/kg) and Zoletil (50 mg/kg) and with additional maintenance of anesthesia using 3% isoflurane, O_2_ (1 liter/min), with continuous vacuum to remove excess isoflurane. Subcutaneous tumors were surgically exposed by a small incision around the tumor, which was further expanded using blunt dissection to separate the epidermis and dermis from the peritoneal wall, thereby creating a skin flap. The skin flap was expanded to a distance from the body suitable for imaging. Once the tumors were surgically exposed, imaging was performed with mice restrained on a heated stage at 37°C and maintained under anesthesia, for a maximum of 40 min. Following imaging, tumors were harvested and formalin-fixed for histological processing.

### Orthotopic injections

For orthotopic survival experiments, NOD.Cg-Prkdc^scid^IL2rg^tm1Wjl^/SzAusb mice were anesthetized [3% isoflurane, O_2_ (1 liter/min), with continuous vacuum to remove excess isoflurane] and TKCC05-Luc [1.5 × 10^4^ in 50 μl of PBS/Matrigel (1:1)] or TKCC10-Luc [1 × 10^6^ in 50 μl of PBS/Matrigel (1:1)] was injected into the pancreas during open laparotomy. Briefly, a left subcostal incision was made through the skin and peritoneum, exposing the pancreas, into which tumor cells were injected using a 29-gauge needle. The peritoneal wall and skin were then individually sutured using Vicryl resorbable sutures and clipped. For analgesia, mice were treated subcutaneously generally with buprenorphine (0.075 mg/kg) and topically with Bupivicaine (5 mg/kg). Treatment started when tumors were both palpable and visible by IVIS monitoring (average flux, 5.5 × 10^8^). Treatment schedules began with twice daily oral gavage of FAKi (10 mg/kg) or vehicle for days 1, 2, and 3. Gemcitabine (70 mg/kg) and Abraxane (30 mg/kg) or saline were administered by intraperitoneal injection on days 6 and 9. After a further 3 days without any treatment, the treatment cycle recommenced. Tumor growth was monitored using IVIS imaging. Experimental endpoint was determined upon development of ascites, overnight weight loss of ≥10% or weight loss of ≥20% over the entirety of the experiment, hunching posture, or signs of pain. At endpoint, animals were euthanized; the pancreatic tumor, liver, lungs, and spleen were harvested; visible metastases were quantified; and tissues were formalin-fixed for histological processing.

### Immunoblot

Cell were rinsed twice in PBS, and lysates were prepared in radioimmunoprecipitation assay protein lysis buffer [50 mM Hepes, 1% Triton X-100, 0.5% sodium deoxycholate, 0.1% SDS, 0.5 mM EDTA, 50 mM NaF, 10 mM NA_3_VO_4_, and 1× protease inhibitor cocktail (Roche)]. Protein concentration was determined by Bradford assay, and the lysate volumes were adjusted accordingly to a final concentration of 1 μg/μl. Protein separation was performed by gel electrophoresis using 4 to 12% or 10% bis-tris protein gels. Separated proteins were transferred onto polyvinylidene difluoride membranes, which were blocked overnight at 4°C in 5% skim milk dissolved in tris-buffered saline (TBS) and 0.1% Tween 20 (TBST). After rinsing with TBST, membranes were incubated overnight at 4°C in primary antibody solutions [TBS/bovine serum albumin (BSA)]. Antibodies and their respective dilutions are provided in Table 1. After rinsing with TBST, membranes were then incubated with horseradish peroxidase (HRP)–linked secondary antibodies (1:5000; diluted in 1% skim milk/TBST; GE Healthcare Limited), for 2 hours at room temperature, and rinsed with TBST. Ultra–enhanced chemiluminescence (ECL) or ECL reagent was used to visualize HRP signal imaged on a FUSION FX (Vilber) or on x-ray film (Fujifilm). Densitometry analysis of protein signal was performed in ImageJ (National Institutes of Health).

**Table 1.** Primary antibodies used for immunoblotting.

**Antibody**	**Company**	**Product**	**Dilution**
Total FAK	BD Biosciences	610088	1:500
pTyr^397^-FAK	Invitrogen	44-625G	1:500
GAPDH (14C10)	Cell Signaling Technology	2118L	1:40,000
b-actin	Sigma-Aldrich	A5441	1:40,000
Merlin (NF2) (D1D8)	Cell Signaling Technology	6995	1:1,000

### Histology, IHC, and immunofluorescence

Organotypic matrices and tumor tissues were fixed in 10% neutral buffered formalin, dehydrated using a graded series of ethanol, and embedded in paraffin. Paraffin sections were cut at a thickness of 4 μm and incubated at 60°C to maximize adhesion to Superfrost Plus slides. Cut sections were deparaffinized in xylene and rehydrated in ethanol (100 to 70%) washes. For hematoxylin and eosin staining, slides were stained on a Leica Autostainer XL. All IHC stains were performed on the Leica BOND RX or the DAKO. All optimization was performed using rabbit (Cell Signaling Technology, 3900s) or mouse (DAKO, X0931) immunoglobulin G (IgG) controls.

For Picrosirius red staining, the sections were dewaxed in xylene and rehydrated in graded ethanol washes. Following hematoxylin counterstaining, sections were stained with 0.02% phosphomolybdic acid and 0.1% Picrosirius red (Polysciences) for fibrillar collagen. Sections were then rinsed in acidified water and dehydrated in graded ethanol before coverslipping. Slides were scanned using an Aperio slide scanner. For tumor sections, analysis of Picrosirius red coverage was analyzed using ImageJ (see the “Picrosirius red coverage” section in Macros), while an in-house MATLAB (MathWorks, USA) script was used to analyze the intensity and coverage of Picrosirius red–stained organotypic matrices.

For IHC on the Leica BOND RX, organotypic matrices and tumors sections were dewaxed using BOND Dewax Solution (Leica, AR2992) on a Leica BOND RX, followed by heat-induced epitope retrieval at 93°C for organotypic matrices and at 100°C for tumor sections, with epitope retrieval solution 2 (pH 9; Leica, AR9640) for 30 min. The details for primary antibody dilution and incubation time are provided in Table 2. IHC staining was carried out on the Leica BOND RX Autostainer, followed by hematoxylin counterstaining on a Leica Autostainer XL and coverslipping on a Leica coverslipper (CV5030). Samples were scanned using an Aperio slide scanner. For KPC tumors, 3,3′-Diaminobenzidine (DAB) coverage of tumor tissue was analyzed using ImageJ (see the “DAB coverage” section in Macros). CC3- and Ki67-stained organotypic matrices were scored manually for positive and negative cells, while tumor sections were analyzed for positive and negative cells using QuPath (*79*).

**Table 2.** Primary antibodies used for IHC, with staining performed on the Leica BOND RX Autostainer.

**Antibody**	**Company**	**Product**	**Dilution**	**Incubation (min)**
Pan-cytokeratin (C-11)	NeoMarkers	149-P	1:200	60
Ki67 (SP6)	Thermo Fisher Scientific	RM-9106-S1	1:500	60
CC3	Cell Signaling Technology	9661	1:200	60
pTyr^397^-FAK	Abcam	ab39967	1:4000	30
Merlin (NF2) (D1D8)	Cell Signaling Technology	6995	1:1000	60
GFP	Invitrogen	A11122	1:1000	60

For staining on the DAKO, sections were deparaffinized in xylene and rehydrated in graded ethanol washes. Antigen retrieval was performed using citrate retrieval solution (pH 6; S1699, DAKO) under pressure at 125°C for 1 min, followed by 30 s at 95°C. Cooled slides were then transferred to a DAKO Autostainer, and IHC staining was performed using the antibodies listed in Table 3 using reagents from the DAKO EnVision system according to the manufacturer’s instructions. Samples were scanned using an Aperio slide scanner, and analysis of DAB coverage of tumor tissue was performed using QuPath (*79*).

**Table 3.** Primary antibodies used for IHC using the DAKO Autostainer.

**Antibody**	**Company**	**Product**	**Dilution**	**Incubation** **(min)**
CD31	Taylor Bio-Medical	DIA-310	1:50	60
Total FAK	BD Biosciences	610088	1:100	60

For immunofluorescence staining of cell polarization assays (scratch wound), paraformaldehyde-fixed cells on glass coverslips were rinsed twice with PBS and incubated in blocking buffer [2.5% BSA (Sigma-Aldrich, A7906), 10% donkey serum (Jackson ImmunoResearch, 017-000-121), and 0.02% glycine (Sigma-Aldrich, G7126)] for 1 hour at room temperature. Cells were then incubated overnight at 4°C in anti–GM-130 (BD Transduction Laboratories, 610823) primary antibody at 1:100 dilution. Cells were rinsed three times in PBS and incubated in Alexa Fluor 488–coupled anti-mouse IgG secondary antibody and tetramethyl rhodamine isothiocyanate (TRITC)–phalloidin at 1:50 (Sigma-Aldrich, P1951) for 2 hours at room temperature. Cells were then rinsed twice with PBS, and nuclei were stained with DAPI (4′,6-diamidino-2-phenylindole; Sigma-Aldrich, D9542) at 1 μg/ml for 15 min, followed by three PBS washes and coverslipping using VECTASHIELD microscope mounting medium (Vector Laboratories, H-1000). Imaging of GM-130 and phalloidin was performed using confocal microscopy.

Dual immunofluorescence staining was performed on 4-μm Formalin-fixed paraffin-embedded (FFPE) KPC tissue sections. Slides were deparaffinized in xylene and rehydrated in graded ethanol washes before antigen retrieval using citrate retrieval solution (pH 6; DAKO, S1699) under pressure at 125°C for 1 min, followed by 30 s at 95°C. Cooled slides were then blocked for 1 hour at room temperature in PBS containing 3% BSA and 5% goat serum. Slides were incubated with primary antibodies diluted in blocking buffer: pTyr^397^-FAK (1:100; Abcam, ab39967), αSMA (1:500; Abcam, ab21027), and E-cadherin (1:100; BD Biosciences, 610181). Secondary antibody staining (1:500; Jackson ImmunoResearch) was performed for 1 hour at room temperature in blocking buffer, followed by nuclear counterstaining with DAPI (1 μg/ml; Sigma-Aldrich, D9542) in PBS. Samples were coverslipped with ProLong Diamond Antifade Mountant (Thermo Fisher Scientific).

### Imaging techniques and data analysis in vitro and in vivo

#### Polarized light imaging of Picrosirius red staining

Polarized light microscopy was performed on fixed, deparaffinized, and rehydrated 4-μm sections stained with 0.1% Picrosirius red (Polysciences, 29401-250). Polarized light signal of fibrillar collagen was taken using an Olympus U-Pot polarizer and an Olympus U-ANT transmitted light analyzer fitted to a DM4000 microscope (Leica). Quantification of birefringent signal was analyzed using ImageJ. Briefly, hue-saturation balance thresholding was applied (high birefringence/red-orange 0>H<29 | 0>S<255 | 70>B<255, medium birefringence/yellow 30>H<44 | 0>S<255 | 70>B<255, and low birefringence/green 45>H<245 | 0>S<255 | 70>B<255). Relative area of fibers was then calculated as percentage of total fibers (0>H<245 | 0>S<255 | 70>B<255).

#### SHG imaging

SHG imaging was performed on an inverted Leica DMS 6000 SP8 confocal microscope with a Titanium-Sapphire femtosecond laser (Coherent Chameleon Ultra II) excitation source, operating at 80 MHz and tuned to a wavelength of 880 or 920 nm. SHG intensity was recorded on an RLD-HyD at 440/20 or 460/50 nm, respectively. For organotypic matrices and CDMs, three representative FOVs (512 pixels × 512 pixels) were imaged over a 3D *z*-stack (80-μm depth with a 2.52-μm step size and 30-μm depth with a 1.26-μm step size, respectively). For tissue sections, five ROIs of deparaffinized and rehydrated 4-μm unstained sections were imaged with a step size of 1.26 μm and 20-μm depth. SHG signal intensity and GLCM correlation were quantified using MATLAB (MathWorks, USA).

#### FUCCI cell cycle reporter imaging

Imaging of the FUCCI cell cycle reporter on pillar plates and on soft/stiff hydrogels was performed on an inverted Leica DMS 6000 SP8 confocal microscope with a 25× 0.95 numerical aperture (NA) water objective. FUCCI was excited with a tunable argon laser at 488 and 514 nm. Fluorescence emission was detected on internal HyDs at 520 to 545 nm and 570 to 600 nm for mAzami Green and mKusabira Orange, respectively. For organotypic matrices, pillar plates, and hydrogels, three ROIs (512 pixels × 512 pixels) were imaged over a 20-μm *z*-stack with a step size of 2.52 μm. Imaging of the FUCCI cell cycle reporter in tissues was performed on an inverted Leica DMS 6000 SP8 confocal microscope with a Titanium-Sapphire femtosecond laser (Coherent Chameleon Ultra II) and a 25× 0.95 NA water objective with the excitation source tuned to a wavelength of 920 nm. The signal was recorded using RLD-HyD detectors (using band-pass emission filters at 460/50 nm for SHG signal, 525/50 nm for mAzami Green, and 585/40 nm for mKusabira Orange). Ten ROIs (512 pixels × 512 pixels) per tumors and 15 ROIs (512 pixels × 512 pixels) per liver were imaged over a 20-μm *z*-stack with a step size of 2.52 μm. 3D maximum projections were then generated using Leica LAS X software and analyzed using ImageJ to quantify percentage of red, green, and yellow nuclei indicative of G_1_-G_0_, S-G_2_-M, or G_1_-S cell cycle phase, respectively.

#### Imaging of cell polarization assays

Imaging of cell polarization assays was performed on a Leica DMI 6000 SP8 Basic Confocal, using 488 and 514 nm for Alexa Fluor 488 and TRITC excitation, respectively. DAPI was excited using a UV laser, and signal was detected using internal photomultiplier tube detectors. Cell polarity was analyzed using ImageJ.

#### FLIM-FRET imaging of the FAK biosensor

For in vitro measurements of FAK activity, 1.5 × 10^5^ TKCC05-FAK or TKCC10-FAK cells were seeded onto CDMs and allowed to adhere for 12 hours before FLIM-FRET imaging on CDMs. Imaging of TIF-FAK cells was performed on days 1, 7, and 12 of matrix contraction within the collagen matrix. For in vivo measurements of Enhanced cyan fluorescent protein (ECFP) fluorescence lifetime in subcutaneous xenografts, KPC-FAK cancer cells were injected into the flank of BALB/c-Fox1nuAusb mice. Two hours after treatment with vehicle or FAKi, tumors were surgically exposed using skin flap surgery. Imaging was performed using an inverted Leica DMS 6000 SP8 confocal microscope with a Titanium-Sapphire femtosecond laser cavity (Coherent Chameleon Ultra II) excitation source tuned to a wavelength of 840 nm for ECFP excitation. Signal was recorded using RLD-HyD detectors (using band-pass emission filters at 435/40 nm for SHG signal and 483/40 nm for FLIM). FLIM data were acquired with a PicoHarp 300 Time-correlated single photon counting (TCSPC) system (PicoQuant), and image stabilization was performed using Galene (*63*). Fifty cells per condition for in vitro assays and five ROIs (512 μm by 512 μm) per tumor in vivo were acquired with a scan speed of 400 Hz, with an acquisition time of 2 min and 30 s and a pixel dwell time of 5 μs. Analysis of ECFP lifetimes was performed using FLIMfit (*80*) by manual selection of single-cell membrane ROIs and recording of the exponential function fit to the fluorescence decay data. Reference lifetimes were calculated using a Chroma slide. Lifetime maps were generated from raw data with a smoothing by a 2 × 2 pixel kernel and application of a standard rainbow look-up table, with blue indicating low ECFP fluorescence lifetime and red indicating high ECFP fluorescence lifetime. The background intensity threshold was set to the average background pixel value for each image to exclude areas without fluorescence lifetime measurement and is shown on the lifetime maps as black.

#### IVIS imaging

Orthotopic tumor growth and metastatic development were monitored via luciferase signal imaging on an IVIS Spectrum (PerkinElmer). Luciferin (150 mg/kg; Gold Biotechnology) was administered by intraperitoneal injection, 4 min before imaging. Anesthetized [2 liters of isoflurane, O_2_ (1 liter/min), with continuous vacuum to remove excess O_2_ and isoflurane] mice were placed on the IVIS stage, exposing the left flank, and the signal was acquired with open filters and small binning. Tumor burden was determined on the basis of total flux.

### Macros

#### DAB coverage

This macro was used to separate DAB-stained area of total and pTyr^397^-FAK–stained sections from hematoxylin counterstain.

//ask user for directory source

dir=getDirectory("Select Directory");

//define and create output directory

saveDir = dir+"Output";

File.makeDirectory(saveDir);

fs=File.separator;

//generation of files for batch processing

list=getFileList(dir);

Array.sort(list);

    for(i=0;i<list.length;i++){

    filename = dir +list[i];

    if(endsWith(filename, "tif")){

          open(filename);

    //store the image name in the nameStore Variable

      nameStore = getTitle();

    //Set the scale for results in um rather than in pixels

      run("Set Scale...", "distance=125 known=50 pixel=1 unit=um global");

    //Split channels based on DAB staining

      run("Colour Deconvolution", "vectors=[H DAB]");

    //Threshold and count the total number of cells in the TotalCells Image

      selectWindow(nameStore + "-(Colour_1)");

      rename("TotalImage");

      setThreshold(0, 220);

      setOption("BlackBackground", true);

      run("Convert to Mask");

      run("Watershed");

      run("Despeckle");

      run("Set Measurements...", "area limit redirect=None decimal=3");

      run("Analyze Particles...", "size=50-Infinity circularity=0.00-1.00 show=Nothing display summarize");

    //Threshold and calculate the area of DAB stain

      run("Set Measurements...", " area display redirect=None decimal=3");

      selectWindow(nameStore + "-(Colour_2)");

      rename("DABImage");

      setThreshold(0, 180);

      setOption("BlackBackground", true);

      run("Despeckle");

      run("Set Measurements...", "area limit redirect=None decimal=3");

      run("Analyze Particles...", "size=50-Infinity circularity=0.00-1.00 show=Masks display summarize");

      run("Measure");

    //Save Overlaid Images of counts

      run("Merge Channels...","c2=TotalImage c1=DABImage ignore");

      selectWindow("RGB");

      rename(nameStore+"-Overview");

      saveAs("Tiff",saveDir+fs+nameStore+" -Summary.tif");

      run("Close");

      run("Close All");

  }

}

      selectWindow("Summary");

      saveAs("Text", dir + nameStore + "Area Summary.xls");

      run("Close");

      selectWindow("Results");

      run("Close");

#### Picrosirius red coverage

This macro was used to quantify total Picrosirius red–stained area of tumor sections and CDMs. For organotypic matrices, the intensity of the stained area was analyzed using an in-house MATLAB (MathWorks, USA) code.

dir = getDirectory("Select Source Directory");

list = getFileList(dir);

Array.sort(list);

    for(i=0; i<list.length; i++){

      filename = dir + list[i];

      if (endsWith(filename, "tif")){

      open(filename);

      nameStore = getTitle();

      run("8-bit");

      setThreshold(0, 165);

      run("Convert to Mask");

      setThreshold(1,255);

      run("Measure");

      saveAs("Tiff", dir+nameStore+" - Thresholded");

      run("Close All");

    }

}

selectWindow("Results");

saveAs("Text", dir+nameStore+" - PicoCoverage.xls");

run("Close");

#### AIG cluster size

This macro was used to quantify the average size of AIG clusters.

dir = getDirectory("Select Source Directory");

list = getFileList(dir);

Array.sort(list);

for(i=0; i<list.length; i++){

    filename = dir + list[i];

    if (endsWith(filename, "tif")){

      open(filename);

//Stores the name of the title of the image. Files that are saved by this

//macro will use the name of the image at the beginning

      nameStore = getTitle();

      dir1 = File.directory;

    //Set scale of image

      run("Set Scale...", "distance=0.0118 known=1 pixel=1 unit=um global");

    //The RGB image is converted to 8-bit greyscale, and then thresholded to select for the positive stain.

      run("8-bit");

      run("Subtract Background...", "rolling=1000 light");

      setAutoThreshold("Default dark");

      run("Threshold...");

      setThreshold(0,170);

      setOption("BlackBackground", true);

      run("Make Binary", "thresholded remaining black");

      setAutoThreshold("Default dark");

      run("Set Measurements...", "area limit redirect=None decimal=3");

      run("Analyze Particles...", "size=500-infinity pixel show=Masks display summarize add");

      saveAs("Tiff", dir+nameStore+" - Thresholded");

    }

}

      selectWindow("Results");

      saveAs("Results", dir+nameStore+" - Area Count.xls");

      selectWindow("Summary");

      saveAs("Summary". dir+nameStore+" - Area Count.xls");

      run("Close All");

#### FUCCI cell cycle analysis

This macro was used to quantify the number of cells in G_1_-G_0_, S-G_2_-M, and G_1_-S phases from overlaid images.

dir=getDirectory("Select Directory");

list=getFileList(dir);

Array.sort(list);

tableTitle="Cell Population";

tableTitle2="["+tableTitle+"]";

      run("Table...", "name="+tableTitle2+" width=600 height=600");

    print(tableTitle2, "\\Headings:Image Name\tGreen Cells\tRed Cells\tYellow Cells\tPercent Green\tPercent Red\tPercent Yellow");

setBatchMode(true); //batch mode on

for(i=0;i<list.length;i++){

  filename = dir +list[i];

  if(endsWith(filename, "tif")){

      open(filename);

      //store the image name in the nameStore Variable

    nameStore = getTitle();

    run("Split Channels");

    selectWindow(nameStore + " (blue)");

    close();

    selectWindow(nameStore + " (green)");

    run("Green");

    rename("SG2");

    run("Subtract Background...", "rolling=50");

    //Adjust threshold and change the numbers below

    setThreshold(25, 255);

    setOption("BlackBackground", true);

    run("Convert to Mask");

    run("Despeckle");

    run("Remove Outliers...", "radius=10 threshold=50 which=Bright");

    run("Watershed");

    setThreshold(30, 255);

    run("Set Measurements...", "area limit redirect=None decimal=3");

    run("Analyze Particles...", "size=20-3000 pixel circularity=0.00-1.00 show=Masks display clear");

    GreenCount= nResults;

    rename("SG2Mask");

    run("Green");

    run("RGB Color");

    selectWindow(nameStore + " (red)");

    run("Red");

    rename("G1");

    run("Subtract Background...", "rolling=100");

    setThreshold(30, 255);

    setOption("BlackBackground", true);

    run("Convert to Mask");

    run("Despeckle");

    run("Remove Outliers...", "radius=5 threshold=50 which=Bright");

    run("Watershed");

    setThreshold(15, 255);

    run("Set Measurements...", "area limit redirect=None decimal=3");

    run("Analyze Particles...", "size=20-3000 pixel circularity=0.00-1.00 show=Masks display clear");

    RedCount= nResults;

    rename("G1Mask");

    run("Red");

    run("RGB Color");

    imageCalculator("Add create", "G1Mask","SG2Mask");

    selectWindow("Result of G1Mask");

    saveAs("Tiff",dir+nameStore+" -Summary.tif");

    rename("Result of G1Mask");

    //Analyse Yellow (overlay) cells

      selectWindow("Result of G1Mask");

      run("Colour Deconvolution", "vectors=[User values] [r1]=-7.063186E-4 [g1]=-7.063186E-4 [b1]=0.9999995 [r2]=0.7071067 [g2]=-4.9944286E-4 [b2]=0.7071067 [r3]=-4.9944286E-4 [g3]=0.7071067 [b3]=0.7071067");

    close();

    close();

    run("8-bit");

    setAutoThreshold("Default dark");

    setThreshold(0,5);

    run("Analyze Particles...", "size=100-Infinity show=Masks display clear");

    YellowCount= nResults;

  GreenPercent=((GreenCountYellowCount)/(GreenCount+RedCount+YellowCount))*100;

  RedPercent = ((RedCount-YellowCount)/(GreenCount+RedCount+YellowCount))*100;

  YellowPercent = (YellowCount/(GreenCount+RedCount+YellowCount))*100;

  print(tableTitle2, nameStore + "\t" + GreenCount + "\t" + RedCount + "\t" + YellowCount + "\t" + GreenPercent + "\t" + RedPercent + "\t" + YellowPercent);

      run("Close All");

    }

}

  setBatchMode(false);

  selectWindow("Results");

  run("Close");

  selectWindow("Cell Population");

  saveAs("Results", dir+"Cell Population.xls");

  run("Close");

## Supplementary Materials

### This PDF file includes:

List of Acronyms Figs. S1 to S9 Legends for movies S1 to S6 Members of the APGI Members of the APMA

## Notes

View/request a protocol for this paper from *Bio-protocol*.
