# Supplementary material for: Intravital imaging technology guides FAK-mediated priming in pancreatic cancer precision medicine according to Merlin status
Source: Sci Adv. 2021 Sep 29;7(40):eabh0363. doi: 10.1126/sciadv.abh0363 (PMC8480933; doi:10.1126/sciadv.abh0363)
Supplement: Supplementary file 1 — List of Acronyms Figs. S1 to S9 Legends for movies S1 to S6 Members of the APGI Members of the APMA [file sciadv.abh0363_sm.pdf]

Science Advances
AAAS

Supplementary Materials for

Intravital imaging technology guides FAK-mediated priming in pancreatic cancer precision medicine according to Merlin status

Kendelle J. Murphy, Daniel A. Reed, Claire Vennin, James R. W. Conway, Max Nobis, Julia X. Yin, Cecilia R. Chambers, Brooke A. Pereira, Victoria Lee, Elysse C. Filipe, Michael Trpceski, Shona Ritchie, Morghan C. Lucas, Sean C. Warren, Joanna N. Skhinas, Astrid Magenau, Xanthe L. Metcalf, Janett Stoehr, Gretel Major, Ashleigh Parkin, Romain Bidanel, Ruth J. Lyons, Anaiis Zaratzian, Michael Tayao, Andrew Da Silva, Lea Abdulkhalek; Australian Pancreatic Genome Initiative (APGI); Australian Pancreatic Cancer Matrix Atlas (APMA), Anthony J. Gill, Amber L. Johns, Andrew V. Biankin, Jaswinder Samra, Sean M. Grimmond, Angela Chou, Jacky G. Goetz, Michael S. Samuel, J. Guy Lyons, Andrew Burgess, C. Elizabeth Caldon, Lisa G. Horvath, Roger J. Daly, Nikolaj Gadegaard, Yingxiao Wang, Owen J. Sansom, Jennifer P. Morton, Thomas R. Cox, Marina Pajic, David Herrmann*, Paul Timpson*

*Corresponding author. Email: p.timpson@garvan.org.au (P.T.); d.herrmann@garvan.org.au (D.H.)

Published 29 September 2021, *Sci. Adv.* **7**, eabh0363 (2021)
DOI: 10.1126/sciadv.abh0363

The PDF file includes:

List of Acronyms
Figs. S1 to S9
Legends for movies S1 to S6
Members of the APGI
Members of the APMA

Other Supplementary Material for this manuscript includes the following:

Movies S1 to S6

List of acronyms

3D	Three-dimensional
AIG	Anchorage Independent Growth
APGI	Australian Pancreatic Genome Initiative
APMA	Australian Pancreatic Cancer Matrix Atlas
CC3	Cleaved Caspase-3
CDM	Cell-derived matrix
ECFP	Enhanced Cyan Fluorescent Protein
ECM	Extracellular Matrix
FACS	Fluorescence-Activated Cell Sorting
FAK	Focal Adhesion Kinase
FAKi	Focal Adhesion Kinase inhibitor
FLIM	Fluorescence Lifetime Imaging Microscopy
FOV	Field of view
FRET	Förster Resonance Energy Transfer
FUCCI	Fluorescent Ubiquitination-based Cell Cycle Indicator
GFP	Green Fluorescent Protein
GLCM	Gray-Level Co-occurrence Matrix
ICGC	International Cancer Genome Consortium
IHC	Immunohistochemistry
IVIS	*In Vivo* Imaging System
KPC	Pdx1-Cre ; LSL-Kras$^{G12D/+}$, LSL-Trp53$^{R172H/+}$
Luc	Luciferase

NF2	Neurofibromatosis type 2 (alternative gene name for Merlin)
PanIN	Pancreatic intraepithelial neoplasia
PC	Pancreatic Cancer
PDAC	Pancreatic Ductal Adenocarcinoma
PDCL	Patient Derived Cell Line
PDX	Patient Derived Xenograft
PTK2	Protein Tyrosine Kinase 2 (alternative gene name for FAK)
pTyr	phosphotyrosine
ROI	Region of interest
SHG	Second Harmonic Generation
shRNA	short hairpin RNA
TIF	Telomerase-Immortalized Fibroblast
TKCC	The Kinghorn Cancer Centre
TMA	Tumor Microarray
TWOMBLI	The Workflow Of Matrix BioLogy Informatics

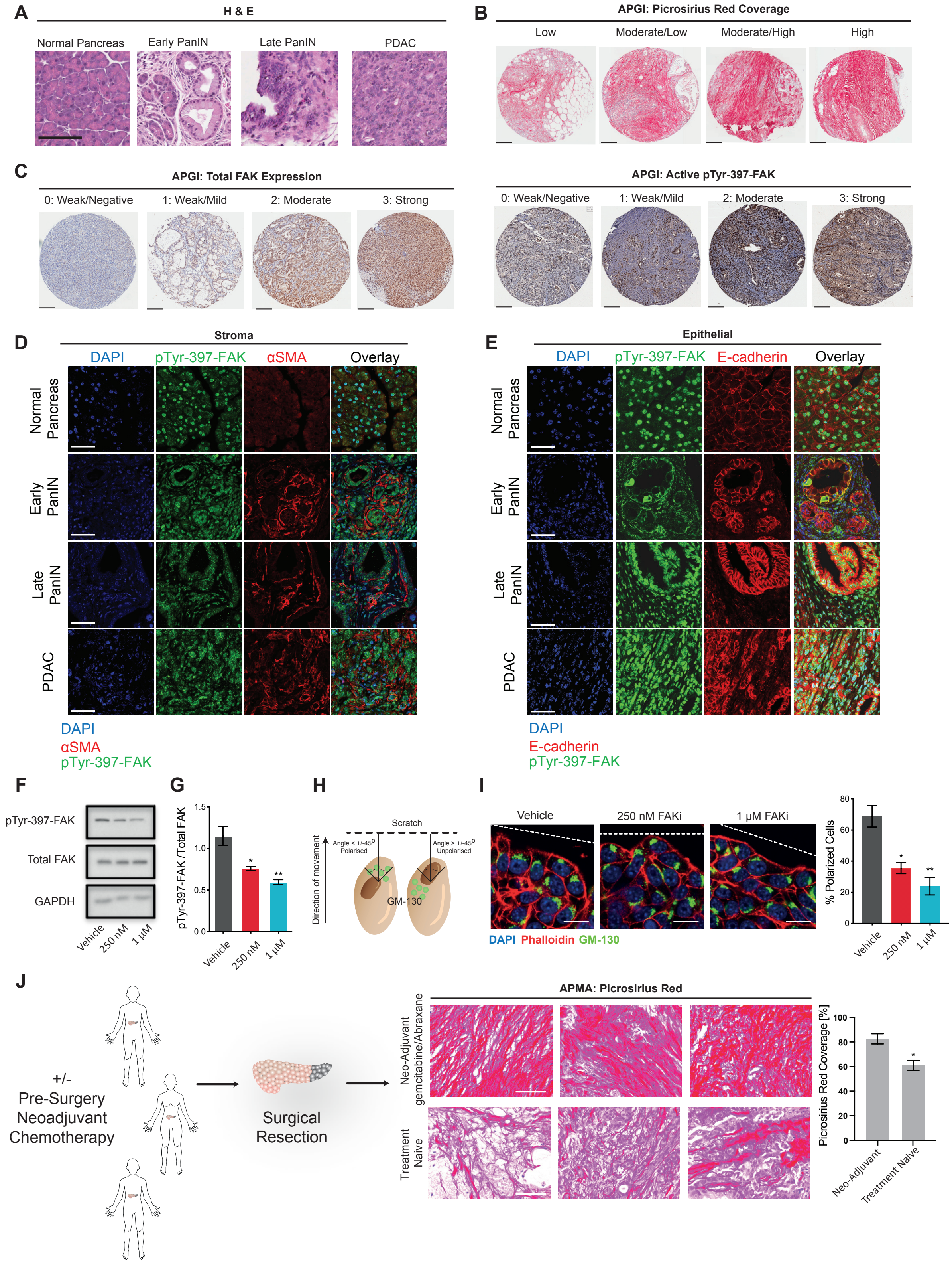

Fig. S1. Epithelial FAK inhibition reduces cell polarization while stromal FAK inhibition

decreases ECM deposition by fibroblasts.

A. Representative H&E Images of Normal Pancreas, Early PanIN, Late PanIN and PDAC, (scale bar, 100 μm). **B.** Representative images of APGI TMA cores stained with picrosirius red showing low, moderate/low, moderate/high and high coverage (scale bar, 200 μm). **C.** Representative images of 0: weak/negative, 1: weak/mild, 2: moderate or 3: strong signal in APGI TMA cores stained with Total FAK (left) and pTyr-397-FAK (right, scale bar, 200 μm). **D,E.** Representative IF images of Normal, Early PanIN, Late PanIN and PDAC KPC tumors stained with DAPI (blue), pTyr-397-FAK (green), αSMA (**D**, red) or E-Cadherin (**E**, red) including overlay (scale bar, 50 μm). **F,G.** Western blot analysis of pTyr-397-FAK and Total FAK in KPC cells treated for 2 hours with vehicle or FAKi. n=3 with sample integrity controls **H.** Schematic representation of cell polarization and Golgi orientation in scratch wound assays, showing examples of a polarized (left) and a non-polarized (right) cell. **I.** Representative images of KPC cell scratch wound assays treated with vehicle or FAKi and stained with anti-GM-130 (green), Phalloidin (red) and DAPI (blue, scale bar, 20 μm), and quantification of cell polarity, showing the percentage of cells with the Golgi orientated to the leading edge of the cell (within 45° of the scratch). n=3 biological repeats with 3 scratch wounds per repeat per condition, and 3 FOV per wound, with ≥ 12 cells per FOV analyzed. Results are mean ± SEM. p-values were determined using an ordinary one-way ANOVA with Tukey correction for multiple comparisons, ns $p>0.05$, * $p<0.05$, ** $p< 0.01$. Unless otherwise stated significance is compared to vehicle **J.** Schematic representation of Human PC surgical resection, representative images and quantification of Picrosirius red stained tumors from treatment naïve patients in the APMA cohort who received no treatment prior to surgery, or neo-adjuvant gemcitabine/Abraxane. n=3 treatment naïve patients, n=4 neo-adjuvant gemcitabine/Abraxane patients, with 5 FOV per tumor. Results are mean ± SEM, p-values were

determined using an unpaired two-tailed *t*-test with Welsh correction for unequal variance, * $p<0.05$.

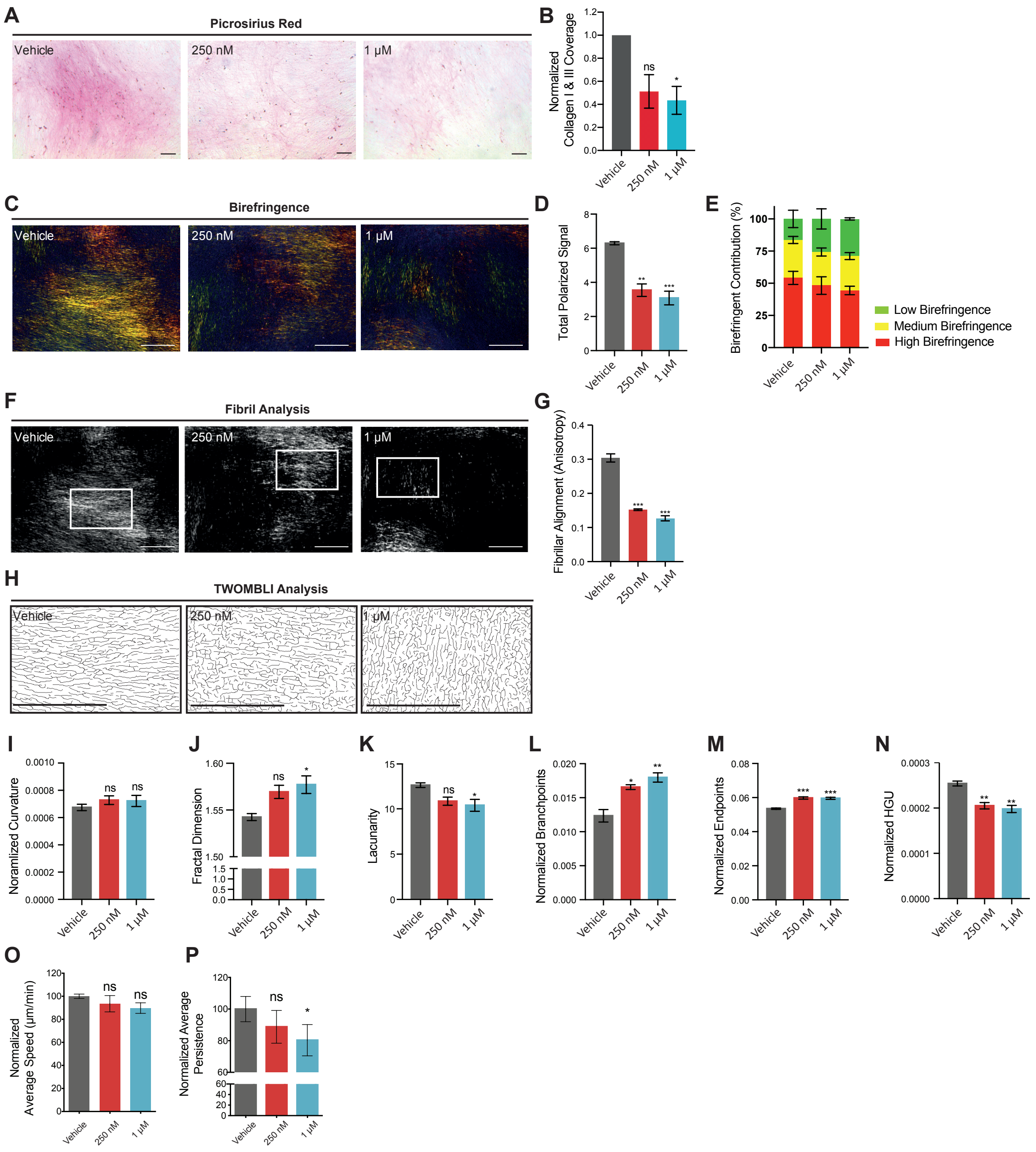

Fig. S2. Stromal FAK inhibition in fibroblast-derived matrices reduces collagen deposition, birefringence and organization

A,B. Representative images of picrosirius red stained CDMs, (scale bar, 100 μm, **A**) and quantification of total collagen I and III coverage (**B**), following treatment with vehicle or FAKi

during CDM production. **C-E.** Representative polarized light images of picrosirius red stained CDMs, (scale bar, 500 μm, **C**) and quantification of total birefringent signal (**D**) and distribution of mature fiber contribution (**E)** of CDMs following treatment with vehicle or FAKi during CDM production. **F,G.** Representative grey-scale images (**F)**, of polarized light images (shown in **C**), and quantification of matrix anisotropy (**G**). **H-N.** Representative binary masks of fibril overlay (**H**) of inset shown in **F.** and quantification of matrix organization following treatment with vehicle (grey), 250 nM (red) or 1 μM (blue) FAKi, including curvature (**I**), fractal dimension (**J**), lacunarity (**K**), normalized branchpoints (**L**), endpoints (**M**) and HGU (**N**); normalization was calculated to average fibril length, to account for changes in matrix quantity. n=3 biological repeats, with 3 matrices per repeat and treatment group, and 5 FOV per matrix. **O,P.** Quantification of individual cell velocity (**O**) and persistence (**P**) on CDMs upon stromal FAK inhibition. n=3 biological repeats with 3 CDMs per condition per repeat, 3 FOV per CDM and 15 cells per FOV. Results are mean ± SEM. p-values were determined using an ordinary one-way ANOVA with Tukey correction for multiple comparisons, ns p>0.05, * p<0.05, ** p< 0.01, *** p<0.001. Unless otherwise stated significance is compared to vehicle.

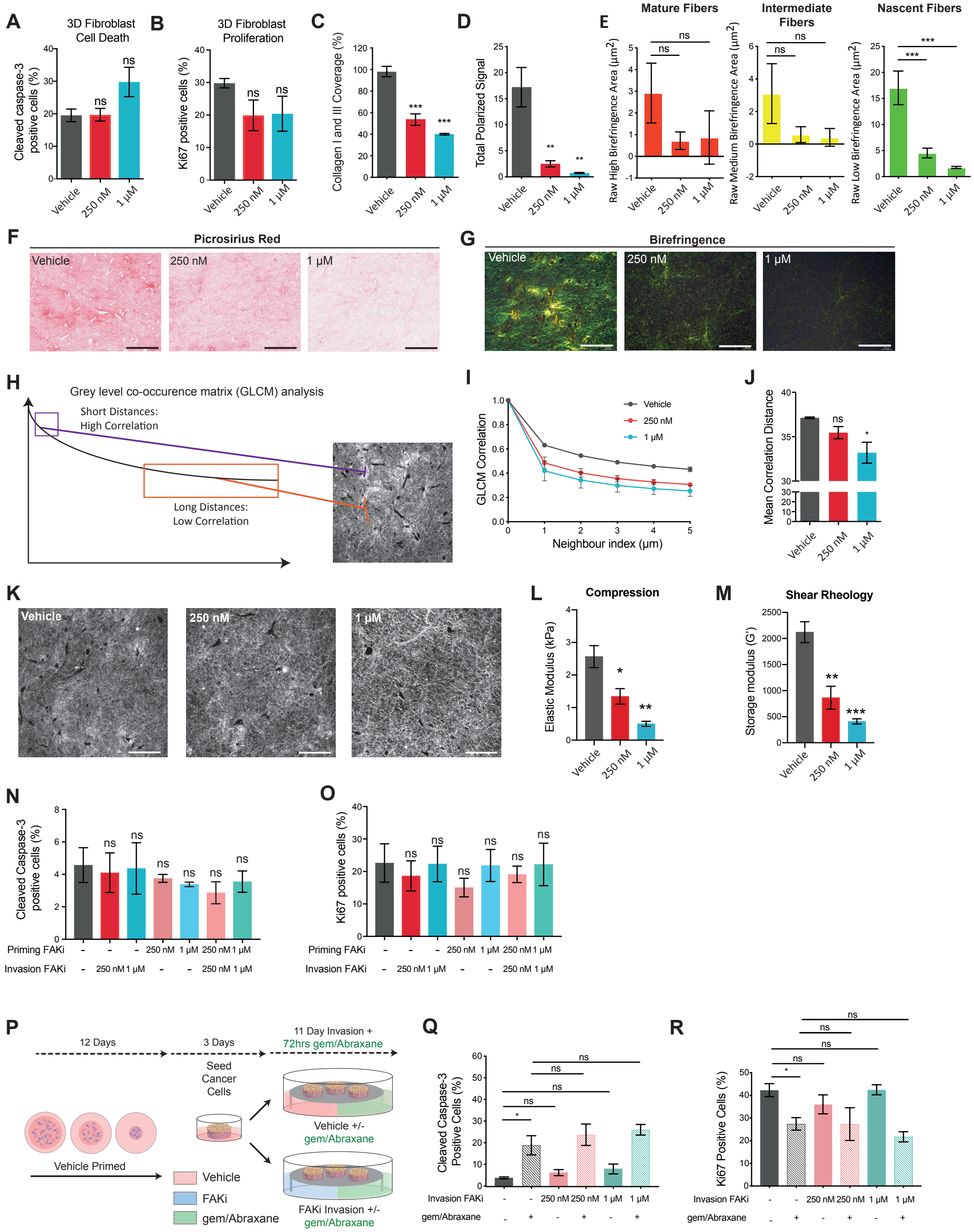
A
3D Fibroblast Cell Death
Cleaved caspase-3 positive cells (%)
B
3D Fibroblast Proliferation
Ki67 positive cells (%)
C
Collagen I and III Coverage (%)
D
Total Polarized Signal
E
Mature Fibers
Intermediate Fibers
Nascent Fibers
Raw High Birefringence Area (μm2)
Raw Medium Birefringence Area (μm2)
Raw Low Birefringence Area (μm2)
Vehicle
250 nM
1 μM
ns
F
Picrosirius Red
G
Birefringence
H
Grey level co-occurence matrix (GLCM) analysis
Short Distances: High Correlation
Long Distances: Low Correlation
I
GLCM Correlation
Neighbour index (μm)
J
Mean Correlation Distance
K
L
Compression
Elastic Modulus (kPa)
M
Shear Rheology
Storage modulus (G')
N
Cleaved Caspase-3 positive cells (%)
O
Ki67 positive cells (%)
Priming FAKi
Invasion FAKi
P
12 Days
3 Days
11 Day Invasion + 72hrs gem/Abraxane
Seed Cancer Cells
Vehicle +/- gem/Abraxane
FAKi Invasion +/- gem/Abraxane
Vehicle Primed
Vehicle
FAKi
gem/Abraxane
Q
Cleaved Caspase-3 Positive Cells (%)
R
Ki67 Positive Cells (%)
Invasion FAKi
gem/Abraxane

Fig. S3. Stromal FAK inhibition in fibroblast-contracted collagen matrices reduces collagen birefringence, organization and stiffness, whilst late FAKi treatment does not improve gemcitabine/Abraxane performance during KPC cell invasion.

A,B. Quantification of cleaved caspase-3 (**A**) and Ki67 (**B**) in TIFs in contracted collagen matrices treated with vehicle or FAKi. **C-E.** Quantification of Collagen I and III coverage (**C**), total polarized light signal (**D**) and high (red), medium (yellow) and low (green) birefringent signal **(E)** of picrosirius red stained collagen matrices treated with vehicle or FAKi, Results are mean ± SD (**E**). **F,G.** Representative bright field images (**F**) and polarized light images (**G**) of picrosirius red stained collagen matrices treated with vehicle or FAKi (scale bar, 100 μm). **H.** Schematic of GLCM analysis of collagen texture, indicating areas of low (red) and high (purple) GLCM correlation. **I,J.** Quantification of GLCM correlation against neighbor index (**I**, μm) and mean correlation distance (**J**) in collagen matrices treated with vehicle or FAKi. **K.** Representative SHG images highlighting fibrillar collagen I organization of collagen matrices treated with vehicle or FAKi (scale bar, 100 μm). **L,M.** Quantification of compression (**L**, bulk modulus) and shear rheology (**M**) of contracted matrices treated with vehicle or FAKi. **N,O.** Quantification of cleaved caspase-3 (**N**) and Ki67 stained KPC cells (**O**) invading into vehicle primed and vehicle treated matrices, vehicle primed matrices treated during invasion with 250 nM FAKi or 1 μM FAKi, 250 nM FAKi or 1 μM FAKi primed matrices, and under chronic treatment with 250 nM FAKi or 1 μM FAKi. **P.** Schematic representation of vehicle primed matrices followed by KPC cell seeding and late epithelial treatment with vehicle or FAKi during cell invasion for 11 days prior to 72 hours of treatment with saline or gemcitabine/Abraxane chemotherapy. **Q.** Quantification of KPC cells positive for cleaved caspase-3 invading into and on top of matrices treated during invasion with vehicle/saline alone (grey), vehicle/gemcitabine/Abraxane alone (grey stripes), 250 nM

FAKi/saline (pink), 250nM FAKi/gemcitabine/Abraxane (pink stripes), 1 μM FAKi/saline (green) or 1 μM FAKi/gemcitabine/Abraxane (green stripes). **R.** Quantification of KPC cells positive for Ki67 invading into and on top of matrices treated during invasion with vehicle/saline alone (grey), vehicle/gemcitabine/Abraxane alone (grey stripes), 250 nM FAKi/saline (pink), 250nM FAKi/gemcitabine/Abraxane (pink stripes), 1 μM FAKi/saline(green) or 1 μM FAKi/gemcitabine/Abraxane (green stripes). For **Q.** and **R.** vehicle/saline and vehicle/gemcitabine/Abraxane are internal controls also utilized in Fig 4. D and E. n=3 biological repeats, with 3 matrices per repeat, and 3 FOV per matrix for quantification. Unless otherwise stated, results are mean ± SEM. p-values were determined using an ordinary one-way ANOVA with Tukey correction for multiple comparisons, ns $p>0.05$, *$p<0.05$, ** $p<0.01$, *** $p<0.001$. Unless otherwise stated significance is compared to control.

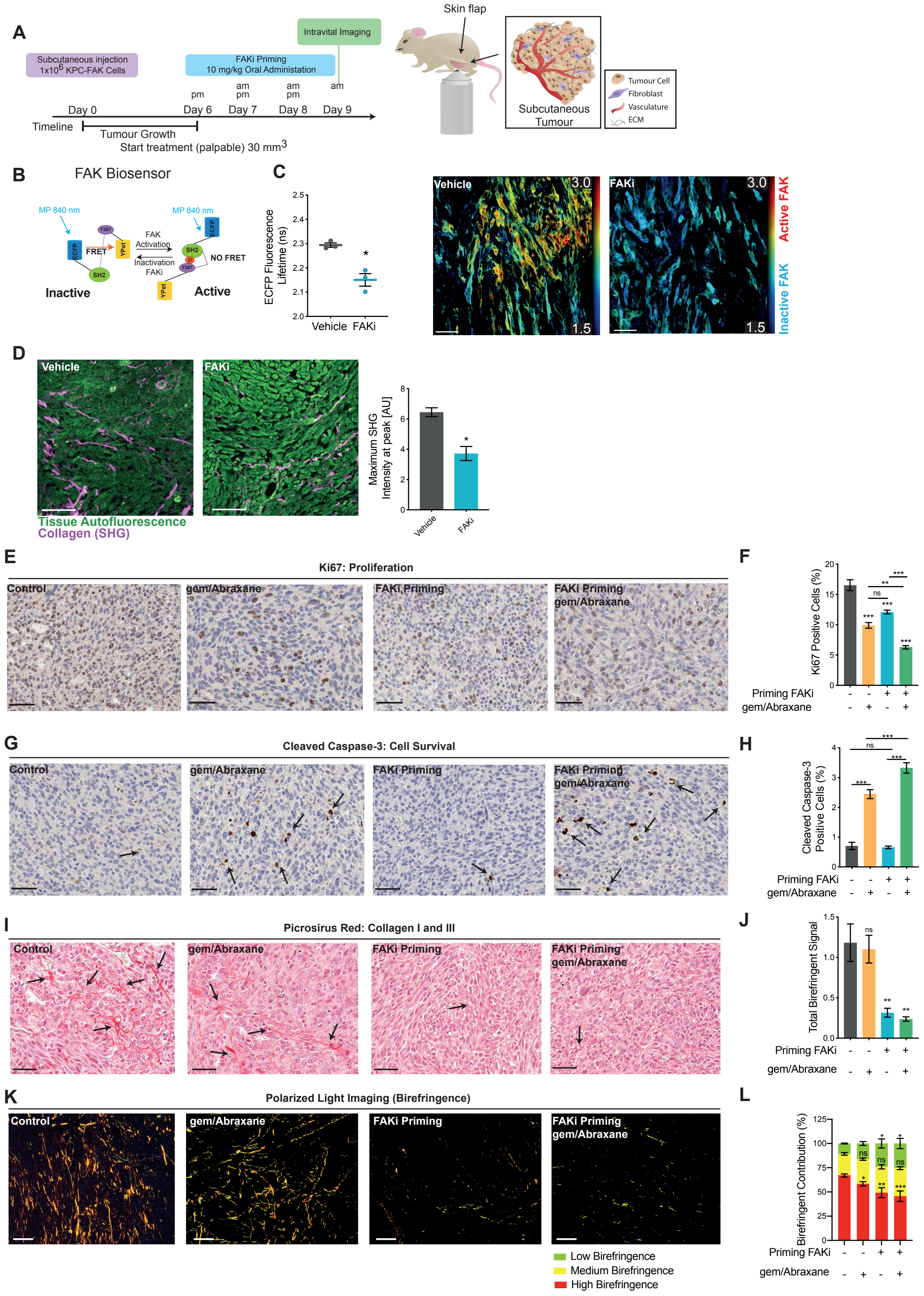

A
Subcutaneous injection 1x10^6 KPC-FAK Cells
FAKi Priming 10 mg/kg Oral Administation
Intravital Imaging
Day 0
Day 6
Day 7
Day 8
Day 9
Timeline
Tumour Growth
Start treatment (palpable) 30 mm3
Skin flap
Subcutaneous Tumour
Tumour Cell
Fibroblast
Vasculature
ECM
B
FAK Biosensor
MP 840 nm
FRET
FAK Activation
Inactivation FAKi
NO FRET
Inactive
Active
C
ECFP Fluorescence Lifetime (ns)
Vehicle
FAKi
Active FAK
Inactive FAK
D
Tissue Autofluorescence
Collagen (SHG)
Maximum SHG Intensity at peak [AU]
E
Ki67: Proliferation
Control
gem/Abraxane
FAKi Priming
FAKi Priming gem/Abraxane
F
Ki67 Positive Cells (%)
Priming FAKi
gem/Abraxane
G
Cleaved Caspase-3: Cell Survival
H
Cleaved Caspase-3 Positive Cells (%)
I
Picrosirus Red: Collagen I and III
J
Total Birefringent Signal
K
Polarized Light Imaging (Birefringence)
Low Birefringence
Medium Birefringence
High Birefringence
L
Birefringent Contribution (%)

Fig. S4. FAKi priming disrupts tumor-ECM feedback by reducing fibrillar collagen content and birefringence while improving gemcitabine/Abraxane performance *in vivo*.

A. Schematic representation of subcutaneous KPC-FAK xenograft establishment, treatment timeline and intravital imaging of surgically exposed tumors to monitor FAK activity. **B,C.** Schematic representation of the FAK biosensor (**B**) and quantification and representative images of ECFP fluorescence lifetime (**C**, nanoseconds, scale bar 50 µm) in mice treated with vehicle or FAKi. n=100 single cells were analyzed per animal (n=3 animals per treatment group). **D.** Representative maximum projection images of SHG signal (magenta) and tissue autofluorescence (green, scale bar, 50 µm) and quantification of peak SHG signal intensity in tumors treated with vehicle or FAKi. 5 FOV per animal, n=8 per treatment group. p-values were determined using an unpaired two-tailed t-test with Welsh correction for unequal variance, ns $p>0.05$, *$p<0.05$. Significance is compared to vehicle. **E,F.** Representative images of Ki67 staining (**E**, scale bar, 50 µm) and quantification of Ki67 positive cells as a percentage of total cells (**F**) in subcutaneous KPC xenograft tumors primed with vehicle or FAKi followed by treatment with saline or gemcitabine/Abraxane. **G,H.** Representative images of cleaved caspase-3 staining, with arrows highlighting positive cells (**G**, scale bar, 50 µm) and quantification of cleaved caspase-3 positive cells as a percentage of total cells (**H**) in subcutaneous KPC xenograft tumors primed with vehicle or FAKi followed by treatment with saline or gemcitabine/Abraxane. **I-L.** Representative images of bright field, (**I,** scale bar, 50 µm) and polarized light microscopy (**K**, birefringence, scale bar, 100 µm, arrows indicate signal) of picrosirius red stained sections with quantification of total picrosirius red birefringent signal (**J**) and contribution to total signal emitted from collagen fibers with high (red), medium (yellow) or low (green) birefringence (**L**) of KPC tumors primed with vehicle or FAKi followed by treatment with vehicle/saline or gemcitabine/Abraxane. n=5 animals

per treatment group and 6 FOV per animal. Results are mean ± SEM. p-values were determined using an ordinary one-way ANOVA with Tukey correction for multiple comparisons, ns $p>0.05$, * $p<0.05$, ** $p<0.01$, *** $p<0.001$. Unless otherwise stated significance is compared to control.

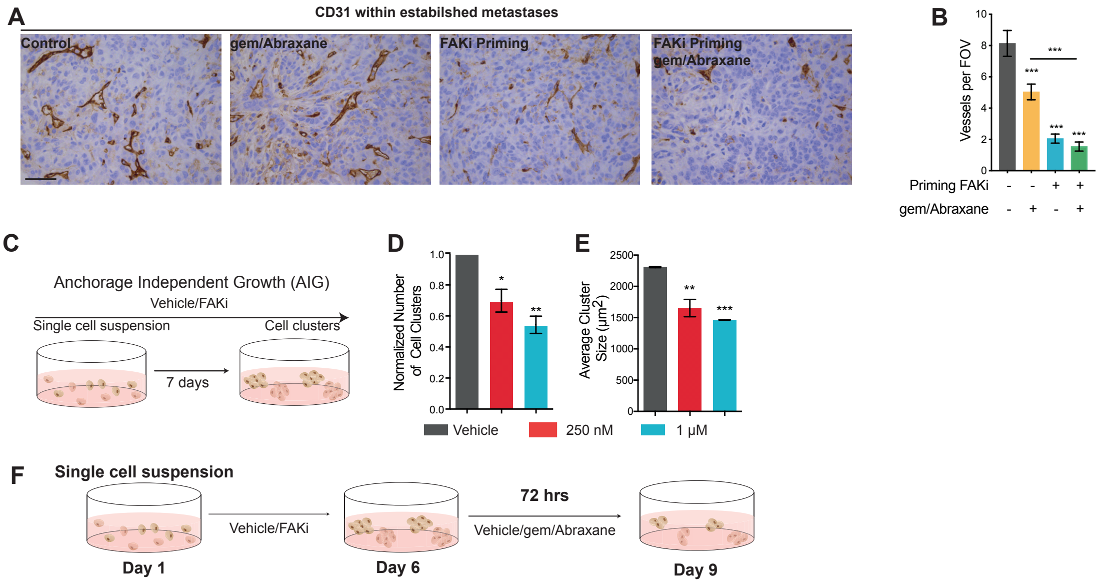

Fig. S5. FAKi priming disrupts the vasculature at secondary PDAC sites and reduces the ability of KPC cells to grow in the absence of cell-environment interaction.

A,B. Representative images of CD31 staining in KPC liver metastases (scale bar, 50 µm, **A**) and quantification of open vessels (**B**) per FOV in mice treated with vehicle/saline (grey, n=8 mice), vehicle/gemcitabine/Abraxane (yellow, n=9 mice), FAKi/saline (blue, n=9 mice) or FAKi/gemcitabine/Abraxane (green, n=10 mice). 8 FOV per animal were analyzed. **C-E.** Schematic representation of an Anchorage Independent Growth (AIG) assay (**C**) and quantification of the normalized number of KPC cell clusters, (**D**) and average cluster size (**E**) following treatment for 7 days with vehicle or FAKi. n=3 biological repeats with three replicates per repeat per treatment group. 10 FOV per replicate were analyzed for cluster size. **F.** Schematic representation of an AIG assay highlighting early vehicle or FAKi treatment followed by 72 hours treatment with saline or gemcitabine/Abraxane. Results are mean ± SEM. p-values were determined using an ordinary one-way ANOVA with Tukey correction for multiple comparisons, * $p<0.05$, ** $p<0.01$, *** $p<0.001$. Unless otherwise stated significance is compared to control.

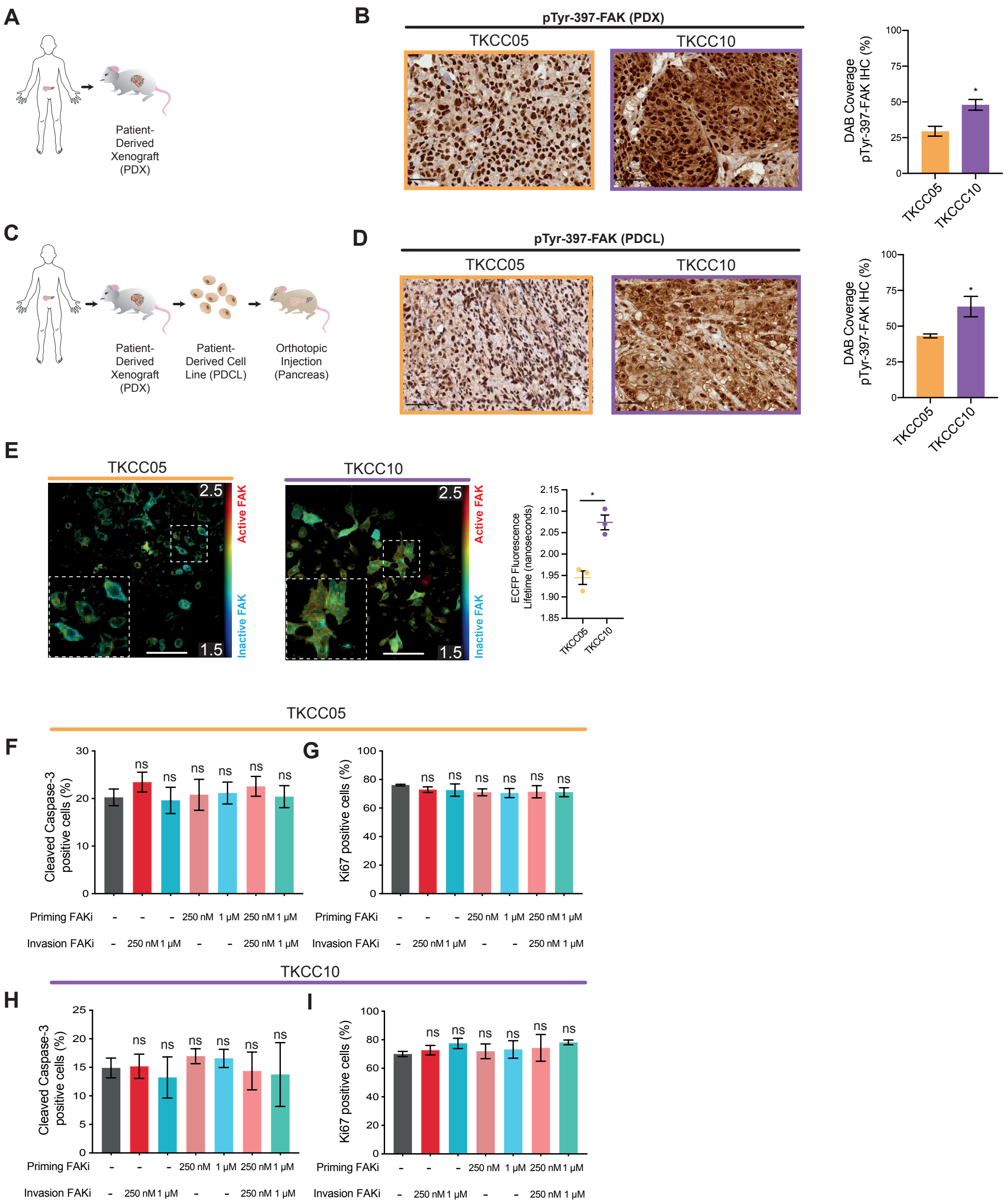

Fig. S6. Validation of total FAK and pTyr397-FAK status in PDAC patient-derived xenografts (PDXs) and cell lines (PDCLs).

A,B. Schematic representation of PDX xenograft establishment (**A**) and representative images and quantification of DAB coverage (**B**) in pTyr-397-FAK stained TKCC05 (orange, left) and TKCC10 (purple, right) PDX tumors (scale bar, 50µm). **C.** Schematic representation of PDCL

generation followed by orthotopic PDCL tumor establishment. **D.** Representative images and quantification of DAB coverage in pTyr-397-FAK stained TKCC05 (orange, left) and TKCC10 (purple, right) PDCL-derived tumors (scale bar, 50 μm). n=3 mice per PDX and PDCL tumor, with 6 FOV per tumor for IHC analysis and DAB quantification. **E.** Representative intensity-merged ECFP fluorescence lifetime maps of FAK activity in TKCC05-FAK and TKCC10-FAK cells (scale bar, 100 μm) and quantification of ECFP fluorescence lifetime. n= 3 biological repeats, with 50 cells per repeat per cell line. Results are mean ± SEM. p-values were determined using an unpaired two-tailed t-test with welsh correction for unequal variance, * $p<0.05$. **F,G.** Quantification of cleaved caspase-3 (**F**) and Ki67 (**G**) stained TKCC05 cells in vehicle primed and vehicle treated matrices (grey), vehicle primed matrices treated during invasion with 250 nM FAKi (red) or 1 μM FAKi (blue), 250 nM FAKi (light pink) or 1 μM FAKi (light blue) primed matrices, and under chronic treatment with 250 nM FAKi (dark pink) or 1 μM FAKi (dark blue). **H,I.** Quantification of cleaved caspase-3 (**H**) and Ki67 (**I**) stained TKCC10 cells in vehicle primed and vehicle treated matrices (grey), vehicle primed matrices treated during invasion with 250 nM FAKi (red) or 1 μM FAKi (blue), 250 nM FAKi (light pink) or 1 μM FAKi (light blue) primed matrices, and under chronic treatment with 250 nM FAKi (dark pink) or 1 μM FAKi (dark blue). n=3 biological repeats, with 3 matrices per repeat, and 3 FOV per matrix for quantification. Results are mean ± SEM. p-values were determined using an ordinary one-way ANOVA with Tukey correction for multiple comparisons, ns $p>0.05$.

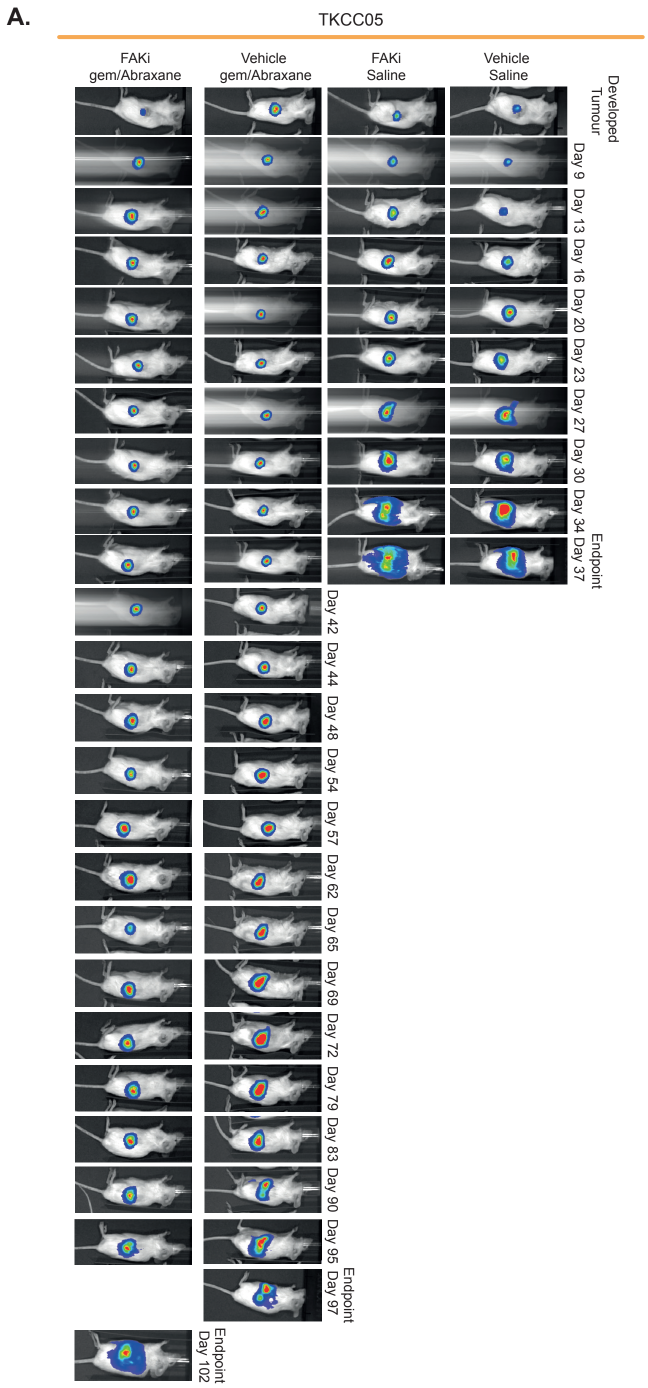

Fig. S7. IVIS monitoring of orthotopic TKCC05 tumor growth.

A. Representative images of whole body IVIS imaging of TKCC05-Luc (Luciferase) orthotopic tumors over time during treatment with vehicle/saline, vehicle/Gemcitabine/Abraxane, FAKi/saline or FAKi/Gemcitabine/Abraxane.

A.

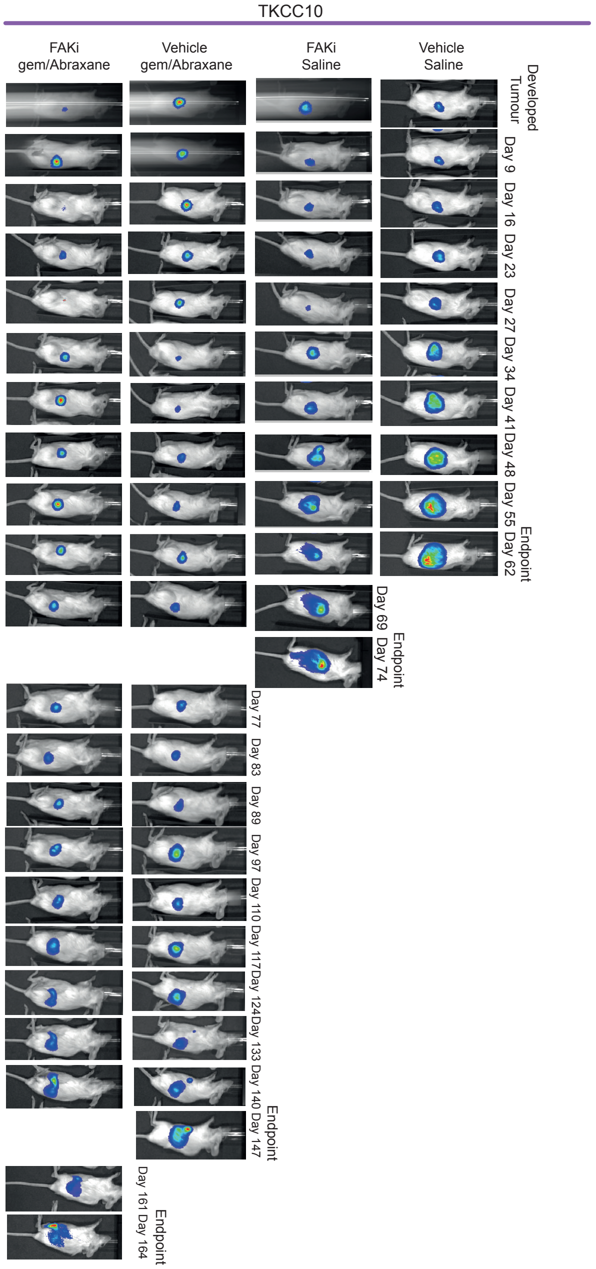

Fig. S8. IVIS monitoring of orthotopic TKCC10 tumor growth.

A. Representative images of whole body IVIS imaging of TKCC10-Luc (Luciferase) orthotopic tumors over time during treatment with vehicle/saline, vehicle/Gemcitabine/Abraxane, FAKi/saline or FAKi/Gemcitabine/Abraxane.

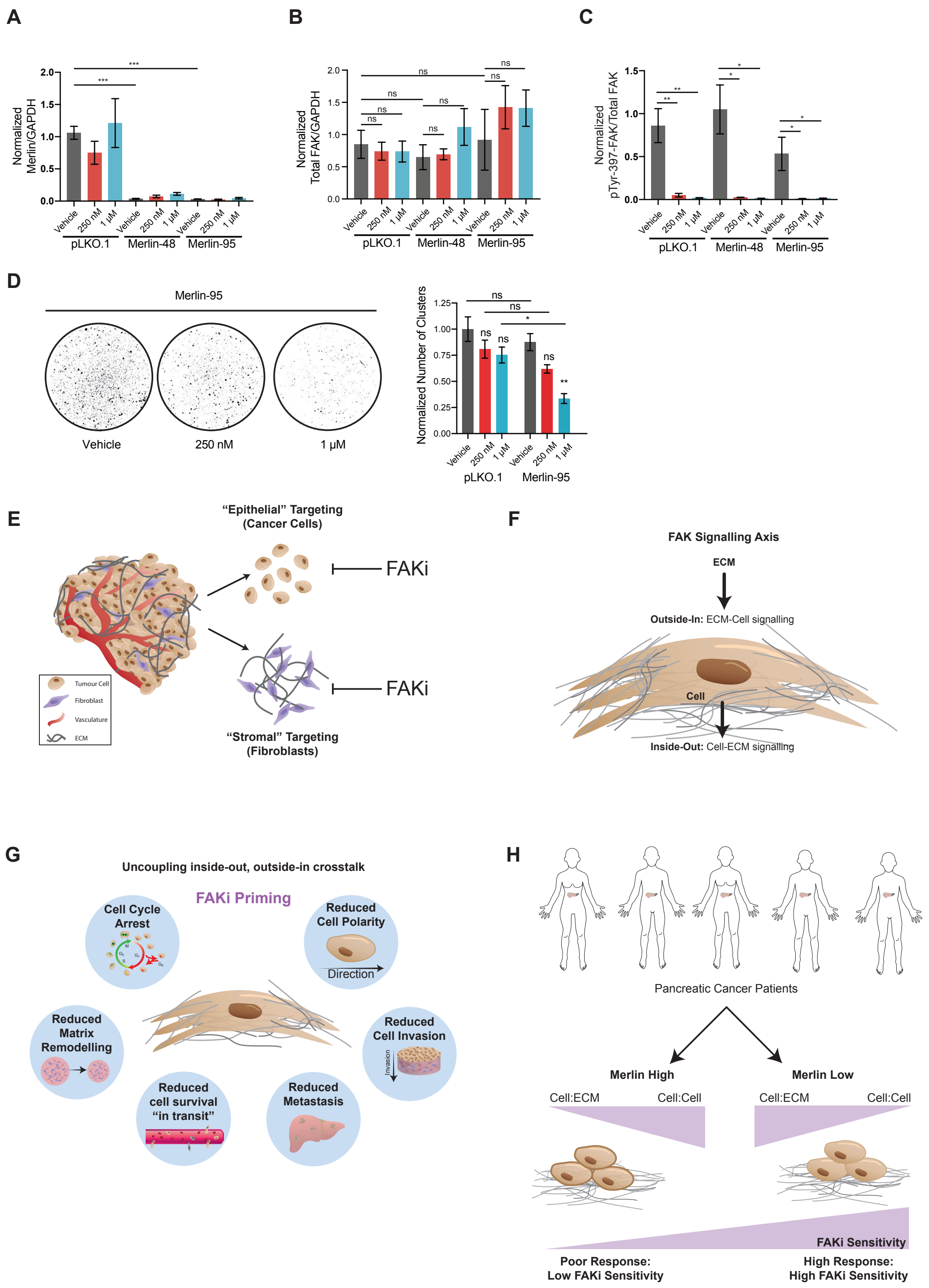

Fig. S9. Merlin knock-down in TKCC05 cells improves sensitivity to FAKi in TKCC05 cells under anchorage-independent conditions.

A-C. Quantification of Western blot analysis in TKCC05 pLKO.1, Merlin-48 and Merlin-95 cell lines on CDMs for Merlin (**A**), Total FAK (**B**) and pTyr-397-FAK levels (**C**) following 2 hours of treatment with vehicle or FAKi. n=3 biological repeats with 1 CDM per condition per repeat. Representative Blot shown in Fig. 8D. **D.** Representative whole well images and quantification of AIG assays of pLKO.1 and Merlin-95 TKCC05 cells treated with vehicle or FAKi for 8 days. For **D.** pLKO.1 is an internal control also utilized in Fig 8. E and F. **E-G.** Schematics of epithelial (cancer cell) and stromal (fibroblast) targeting (**E**), outside-in and inside-out FAK signaling axis **(F)**, the effects we observed upon uncoupling inside-out, outside-in crosstalk *via* FAKi priming **(G)** and stratification of PC patients for FAKi priming according to their Merlin status (**H**). n=3 biological repeats with four technical replicates per treatment per repeat. Results are mean ± SEM. p-values were determined using an ordinary one-way ANOVA with Tukey correction for multiple comparisons, ns $p>0.05$, * $p<0.05$, ** $p< 0.01$, *** $p< 0.001$.

Other Supplementary Materials for this manuscript include the following:

Movie S1. KPC cell adhesion, migration and streaming on CDMs upon late epithelial treatment with vehicle or FAKi (scale bar, 200 μm). Representative images from this Movie 72 hours post-seeding are shown in Fig. 2E.

Movie S2. KPC cell adhesion, migration and streaming on CDMs upon early stromal priming with vehicle or FAKi (scale bar, 200 μm). Representative images from this Movie 72 hours post-seeding are shown in Fig. 2L.

Movie S3. FAKi priming reduces collagen crosslinking visualized by Second Harmonic Generation (SHG) imaging (scale bar, 100 μm).

Movie S4. FAKi reduces FAK activity *in vivo* shown by FLIM-FRET imaging of the FAK biosensor in live tumors (scale bar, 50 μm).

Movie S5. FAKi priming prior to chemotherapy increases the number of cells in S-G2/M phase in live KPC tumors (scale bar, 50 μm).

Movie S6. FAKi priming prior to chemotherapy increases the number of cells in S-G2/M phase in residual metastases at secondary sites (scale bar, 50 μm).

Members of the APGI:

Garvan Institute of Medical Research Amber L. Johns[1], Anthony J Gill[1, 5], Lorraine A. Chantrill[1,22], Paul Timpson[1,] Angela Chou[1,5], Marina Pajic[1], Tanya Dwarte[1], David Herrmann[1], Claire Vennin[1], Thomas R Cox[1], Brooke Pereira[1], Shona Ritchie[1], Daniel A Reed[1], Cecilia R Chambers[1], Xanthe Metcalf[1], Max Nobis[1], Gloria Jeong[1], Lara Kenyon[1]. **QIMR Berghofer Medical Research Institute** Nicola Waddell[2], John V. Pearson[2], Ann-Marie Patch[2], Katia Nones[2], Felicity Newell[2], Pamela Mukhopadhyay[2], Venkateswar Addala[2], Stephen Kazakoff[2], Oliver Holmes[2], Conrad Leonard[2], Scott Wood[2]. **University of Melbourne, Centre for Cancer Research** Sean M. Grimmond[3], Oliver Hofmann[3]. **Royal North Shore Hospital** Jaswinder S. Samra[5], Nick Pavlakis[5,] Jennifer Arena[5], Hilda A. High[5]. **Bankstown Hospital** Ray Asghari[6], Neil D. Merrett[6], Amitabha Das[6]. **Liverpool Hospital** Peter H. Cosman[7], Kasim Ismail[7]. **St Vincent's Hospital** Alina Stoita[8], David Williams[8], Allan Spigellman[8]**. Westmead Hospital** Duncan McLeod[9], Judy Kirk[9]. **Royal Prince Alfred Hospital, Chris O'Brien Lifehouse** James G. Kench[10], Peter Grimison[10], Charbel Sandroussi[10], Annabel Goodwin[7,10]. **Prince of Wales Hospital** R. Scott Mead[1,11], Katherine Tucker[11], Lesley Andrews[11]. **Fiona Stanley Hospital** Michael Texler[12], Cindy Forrest[12], Mo Ballal[12,13], David Fletcher[12]**. St John of God Healthcare** Maria Beilin[13], Kynan Feeney[13] Krishna Epari[13] Sanjay Mukhedkar[13]. **Epworth HealthCare** Nikolajs Zeps[23]. **Royal Adelaide Hospital** Nan Q Nguyen[14], Andrew R. Ruszkiewicz[14], Chris Worthley[14]. **Flinders Medical Centre** John Chen[15], Mark E. Brooke-Smith[15], Virginia Papangelis[15]. **Envoi Pathology** Andrew D. Clouston[16]. **Princess Alexandra Hospital** Andrew P. Barbour[17], Thomas J. O'Rourke[17], Jonathan W. Fawcett[17], Kellee Slater[17], Michael Hatzifotis[17], Peter Hodgkinson[17]. **Austin Hospital** Mehrdad Nikfarjam[18]. **Johns Hopkins Medical Institutes** James R. Eshleman[19], Ralph H. Hruban[19], Christopher L. Wolfgang[19]. **ARC-Net Centre for**

Applied Research on Cancer Aldo Scarpa[20], Rita T. Lawlor[20], Vincenzo Corbo[20], Claudio Bassi[20]. **University of Glasgow** Andrew V Biankin [21], Nigel B. Jamieson[21] David K. Chang[1, 21], Stephan B. Dreyer[21].

[1]The Kinghorn Cancer Centre, Garvan Institute of Medical Research, 370 Victoria Street, Darlinghurst, Sydney, New South Wales 2010, Australia.

[2]QIMR Berghofer Medical Research Institute, 300 Herston Rd, Herston, Queensland 4006, Australia.

[3]University of Melbourne, Centre for Cancer Research, Victorian Comprehensive Cancer Centre, 305 Grattan Street, Melbourne, Victoria 3000, Australia.

[4] Institute for Molecular Bioscience, University of QLD, St Lucia, Queensland 4072, Australia.

[5]Royal North Shore Hospital, Westbourne Street, St Leonards, New South Wales 2065, Australia.

[6]Bankstown Hospital, Eldridge Road, Bankstown, New South Wales 2200, Australia.

[7]Liverpool Hospital, Elizabeth Street, Liverpool, New South Wales 2170, Australia.

[8] St Vincent's Hospital, 390 Victoria Street, Darlinghurst, New South Wales, 2010 Australia.

[9]Westmead Hospital, Hawkesbury and Darcy Roads, Westmead, New South Wales 2145, Australia.

[10]Royal Prince Alfred Hospital, Missenden Road, Camperdown, New South Wales 2050, Australia.

[11]Prince of Wales Hospital, Barker Street, Randwick, New South Wales 2031, Australia.

[12]Fremantle Hospital, Alma Street, Fremantle, Western Australia 6959, Australia.

[13] St John of God Healthcare, 12 Salvado Road, Subiaco, Western Australia 6008, Australia.

[14] Royal Adelaide Hospital, North Terrace, Adelaide, South Australia 5000, Australia.

[15] Flinders Medical Centre, Flinders Drive, Bedford Park, South Australia 5042, Australia.

[16] Envoi Pathology, 1/49 Butterfield Street, Herston, Queensland 4006, Australia.

[17] Princess Alexandra Hospital, 199 Ipswich Rd, Woolloongabba QLD 4102

[18] Austin Hospital, 145 Studley Road, Heidelberg, Victoria 3084, Australia.

[19] Johns Hopkins Medical Institute, 600 North Wolfe Street, Baltimore, Maryland 21287, USA.

[20] ARC-NET Center for Applied Research on Cancer, University of Verona, Via dell'Artigliere, 19 37129 Verona, Province of Verona, Italy.

[21] Wolfson Wohl Cancer Research Centre, Institute of Cancer Sciences, University of Glasgow, Garscube Estate, Switchback Road, Bearsden, Glasgow, Scotland G61 1BD, United Kingdom.

[22] Wollongong Hospital, Illawarra and Shoalhaven Local Health District, Loftus Street, Wollongong NSW 2500.

[23] Epworth HealthCare, 89 Bridge Rd, Richmond VIC 3121, Australia

Members of the APMA

Paul Timpson[1], Thomas R. Cox[1], Marina Pajic[1], Anthony J. Gill[1,2], Jaswinder Samra[1,2], Brooke A. Pereira[1], David Herrmann[1], Amber L. Johns[1], Gloria Jeong[1], Shona Ritchie[1], Daniel A. Reed[1], Cecilia R. Chambers[1], Janett Stoehr[1], Morghan C. Lucas[1], Joanna N. Skhinas[1], Lea Abdulkhalek[1], Max Nobis[1], Tatjana Schmitz[1], Victoria Lee[1], Xanthe L. Metcalf[1], Sean M Grimmond[3], Kym Pham Stewart[3], Mehreen Arshi[1], Angela M Steinmann[1], Lara Kenyon[1]

[1]The Kinghorn Cancer Centre, Garvan Institute of Medical Research, 370 Victoria Street, Darlinghurst, Sydney, New South Wales 2010, Australia.

[2]Royal North Shore Hospital, Westbourne Street, St Leonards, New South Wales 2065, Australia.

[3]University of Melbourne Centre for Cancer Research, Victorian Comprehensive Cancer Centre, 305 Grattan Street, Melbourne, Victoria, 3000, Australia
